# Genome-wide identification of the tea plant bHLH transcription factor family and discovery of candidate regulators of trichome formation

**DOI:** 10.1038/s41598-021-90205-7

**Published:** 2021-05-24

**Authors:** Renjian Liu, Yuyuan Wang, Song Tang, Jiarong Cai, Shaoqun Liu, Peng Zheng, Binmei Sun

**Affiliations:** College of Horticulture, South China Agricultural University, Guangzhou, Guangdong 510642 People’s Republic of China

**Keywords:** Computational biology and bioinformatics, Genetics, Molecular biology

## Abstract

Leaf trichomes play vital roles in plant resistance and the quality of tea. Basic helix-loop-helix (bHLH) transcription factors (TFs) play an important role in regulating plant development and growth. In this study, a total of 134 CsbHLH proteins were identified in the *Camellia sinensis* var. *sinensis* (CSS) genome. They were divided into 17 subgroups according to the *Arabidopsis thaliana* classification. Phylogenetic tree analysis indicated that members of subgroups IIIc-I and IIIc-II might be associated with trichome formation. The expression patterns of *CsbHLH116*, *CsbHLH133*, *CsbHLH060*, *CsbHLH028*, *CsbHLH024*, *CsbHLH112* and *CsbHLH053* from clusters 1, 3 and 5 were similar to the trichome distribution in tea plants. CsbHLH024 and CsbHLH133 were located in the cell nucleus and possessed transcriptional activation ability. They could interact with CsTTG1, which is a regulator of tea trichome formation. This study provides useful information for further research on the function of CsbHLHs in trichome formation.

## Introduction

Trichomes are developed from epidermal cells and mainly distributed on the undersurface of plant leaves^1–4^. Plant leaf trichomes are an important basis for botanical classification and play key roles in plant resistance to biotic and abiotic stresses. According to the morphology and function of trichomes, they are classified as nonbranched or branched trichomes and nonglandular or glandular trichomes^5–8^. Glandular trichomes protect plants from herbivores and insects by accumulating and secreting a series of secondary metabolites, such as alkaloids, nicotine and terpenes^9,10^. Nonglandular trichomes can enhance plant tolerance in response to extreme temperatures, drought and ultraviolet radiation^11–14^. Trichome formation has been systematically investigated in *Arabidopsis thaliana*, *Solanum lycopersicum*, *Cucumis sativus* L., *Oryza sativa* L., *Nicotiana tabacum* L., *Gossypium* spp. and *Glycine max*^15–21^. Trichome formation is induced by cell differentiation. *Arabidopsis thaliana* trichome development is an ideal model for the study of cell differentiation^22^. Many transcription factors (TFs) are associated with trichome development in *Arabidopsis thaliana,* including R2R3-MYB TFs, bHLH TFs and WD40-repeat (WDR) proteins^23–25^. The MYB-BHLH-WDR (MBW) complex can positively regulate *Arabidopsis thaliana* trichome formation^26^. In addition, most dicots possess a similar regulatory mechanism of trichome formation^27^.

Tea, one of the three major nonalcoholic beverages, possesses high nutritional and health-benefitting properties^28,29^. Tender leaves serving as the main raw material are used for tea production. Apical buds and young leaves possess trichomes in most tea plant cultivars; thus, leaf trichomes have become a critical diagnostic characteristic in tea taxonomy. Abundant trichomes are generally indicate high quality in Chinese tea. An abundance of trichomes on tea products indicates that they were processed using tender leaves of tea plants^8^. Tea trichomes contain abundant metabolites, including theanine, catechins, volatiles and caffeine^30,31^. These metabolites have different flavors and tastes in tea infusions. Theanine makes the flavor of sweet and umami, and catechins and caffeine make the flavor of bitterness and astringency in tea infusions^30^. Tea trichomes also possess high contents of benzoic acid derivatives, lipid oxidation derivatives and monoterpene derivatives, which contribute to tea flavor and aroma^32^. In addition, some signaling genes related to diseases and anti-herbivore and anti-abiotic peptides were specifically transcribed in tea trichomes^32^.

Basic helix-loop-helix (bHLH) TFs are the second-largest TF family in plants^33^. Their conserved domains contain two different functional regions, a basic region and a helix-loop-helix (HLH) region, which are composed of 50–60 amino acids^34–36^. The basic region in the N-terminal domain consists of 13–17 amino acids and binds to the consensus hexanucleotide E-box (CANNTG). The HLH region in the C-terminal domain includes approximately 40 amino acids and contributes to the formation of homodimeric complexes and heterodimeric complexes, as well as the promotion of interactions with other TFs^37–40^. bHLH TFs play important roles in responses to stresses, secondary metabolism biosynthesis and plant growth and development^34,41–44^. Numerous studies have demonstrated that bHLH TFs play a critical role in trichome formation. The bHLH proteins GLABRA3 (GL3) and ENHANCER OF GLABRA3 (EGL3) are important for the regulation of trichome formation in *Arabidopsis thaliana*^45,46^. GL3 and EGL3 interact with the WDR and R2-R3 MYB proteins to induce trichome formation by targeting *GLABRA2 (GL2)* transcription^47–49^. GL3 also facilitates trichome branching formation by positively regulating *FURCA4 (FRC4)* expression^50^. Trichome formation in tomato plants is independent of SlGL3^51^. Trichome formation is extremely complex in tea plants. Whether bHLH TFs are related to trichome formation in tea plants is less well understood.

In this study, the bHLH family was identified in *Camellia sinensis* var. *sinensis* (CSS) genome, and characteristic analyses were systematically performed. The results of phylogenetic tree and expression pattern analyses showed that *CsbHLH024* and *CsbHLH133* might be associated with tea trichome formation. They were further selected for subcellular localization, transcriptional activation and yeast two-hybrid (Y2H) assays, aiming to preliminarily determine their function. This study provides useful information for further research on the function of CsbHLH TFs in the regulation of trichome formation.

## Materials and methods

### Identification of the bHLH gene family in tea plants

bHLH TF sequences were acquired from the Tea Plant Information Archive (TPIA) (http://tpia.teaplant.org)^31^. The SMART database^52^, National Center for Biotechnology Information (NCBI) conserved domain search service^53^ and hidden Markov model (HMM) profile of the bHLH domain (PF00010) in the Pfam database^54^ were used to filter redundant bHLH proteins in tea plants. The full-length amino acid sequences, molecular weights (Mws), theoretical isoelectric points (pIs) and instability index values of these proteins were predicted using the ExPASy server^55^. The CsbHLHs were renamed *CsbHLH001* to *CsbHLH134* based on the gene ID order.

### Conserved motif and gene structure characterization

The conserved motifs of the bHLH protein were identified using Multiple EM for Motif Elicitation (MEME) with the following parameter settings: site distribution, any number of repetitions; number of motifs, 15; maximum motif width, 100; minimum motif width, 6; maximum number of sites, 100; and minimum number of sites, 5^56^. Both coding DNA sequences (CDSs) and genomic sequences of bHLHs were used for determination of the gene structure with TBtools software^57^.

### Phylogenetic tree analysis

The bHLH protein sequence of *Arabidopsis thaliana* (128) was downloaded from The *Arabidopsis* Information Resource (TAIR) database^58^, and those of *Oryza sativa* L. (144) and *Actinidia chinensis* (164) were acquired from the Plant TF Database version 4.0^59^. All sequences were renamed and listed in Table S4. Moreover, the conserved bHLH domains of these proteins were subjected to multiple sequence alignment using ClustalX 2.1 with the default parameters. MEGA X was used to construct neighbor-joining phylogenetic trees with the following parameters: 1000 bootstrap replications; Poisson model; and pairwise deletion^60^. The phylogenetic trees were optimized using Evolview v3^61^.

### Transcriptome data analysis

The transcriptome data of tea plant *bHLHs* were obtained from TPIA^31^. The expression patterns of *CsbHLHs* in different developmental leaf tissues, including apical buds, young leaves, mature leaves and old leaves, were determined with R Language software.

### Plant material

‘Fenghuangdancong’ (‘FHDC’), ‘Renhuabaihao’ (‘RHBH’), ‘Yinghongjiuhao’ (‘YH9’) and ‘Baiyedancong’ (‘BYDC’) were cultivated at South China Agricultural University (Guangzhou, China). According to institutional, national and international guidelines, the material used for research purposes does not require specific permissions. The use of rights to these plant materials was obtained by our lab. Apical buds, young leaves, mature leaves and old leaves of the four tea plant cultivars were collected. All the samples were collected in three biological replicates, with two technical replicates for each biological replicate. Some samples were used to conduct trichome observations by stereoscopy (Carl Zeiss, Germany), and the other samples were used for RNA extraction. The samples used for RNA extraction were immediately frozen in liquid nitrogen and stored at -80℃.

### qRT-PCR analysis

The total RNA of the tea plant samples was extracted and isolated using the HiPure Total RNA kit (R4111, Magen, China). RNA reverse transcription was carried out with the HiScript III RT SuperMix for qPCR Reagent Kit with gDNA Wiper (R323-01, Vazyme, China). The primers employed for qPCR were designed with the NCBI Primer design tool (https://www.ncbi.nlm.nih.gov/tools/primer-blast/index.cgi?LINK_LOC=BlastHome). All primers are listed in Table S5. *SAND1* was used as the reference gene. qRT-PCR analysis was performed with the Bio-Rad CFX384 Touch TM system following routine procedures (Bio-Rad, Hercules, CA, USA)^55^. Relative expression was calculated using the 2^−ΔΔCt^ method^62^. All samples were analyzed in three biological replicates, with three technical replicates for each biological replicate.

### Subcellular localization

The CDSs of *CsbHLH133* and *CsbHLH024* without the termination codon were cloned into the pEAQ-EGFP vector. The recombinant plasmids and nuclear localization signal (NLS-DsRed) were transformed into *Agrobacterium tumefaciens* strain GV3101, which was mixed and injected into tobacco (*Nicotiana benthamiana*) leaves. After 48 h, the tobacco leaves were collected for fluorescence microscopy observations (Carl Zeiss, Germany).

### Dual-luciferase reporter assay

A dual-luciferase reporter assay system (Promega, USA) was used for the determination of transcriptional activation. The full-length CDSs of *CsbHLH133* and *CsbHLH024* were ligated into the pEAQ-PBD vector and fused with the GAL4 DNA-binding domain under the control of the CaMV 35S promoter. The empty vector, the reporter gene (GAL-LUC) and the generated constructs were transformed into the GV3101 *Agrobacterium* strain. The *Agrobacterium* strain containing the empty vector or the constructs and the reporter was coinfiltrated into tobacco leaves. After three days, the tobacco leaves were collected, and the activity of *Renilla* LUC/firefly was measured according to a previously described protocol^44^.

### Construction of the potential regulatory network

The protein sequences of TFs associated with *Arabidopsis thaliana* trichome formation, including MYB23, triptychon (TRY), GL3, ENHANCER of TRY and CPC1 (ETC1), GLABRA1 (GL1), GL2, CAPRICE (CPC), TRANSPARENT TESTA GLABRA1 (TTG1), ENHANCER of TRY and CPC2 (ETC2), SENSITIVE TO ABA AND DROUGHT2 (SAD2) and EGL3^63^, were obtained from the TAIR database^58^. They were submitted to the STRING server for protein–protein interaction network functional enrichment analysis with the default parameters (https://string-db.org).

### Yeast two-hybrid assay

The full-length CDSs of *CsbHLH024*, *CsbHLH133* and *CsTTG1* were separately cloned into the pGBKT7 and pGADT7 vectors via one-step cloning (C112, Vazyme, China). The resulting positive, negative and recombinant plasmids were subsequently transformed into a Y2HGold yeast strain (YC1002, Weidi Biotechnology, China). A Y2H assay was then performed according to the manufacturer’s instructions (Clontech), and image acquisition was performed via Adobe Illustrator CS2020 (Germany, Zeiss). All primers used are listed in Table S5.

## Results and analysis

### Identification and conserved domain analysis of *CsbHLHs*

A total of 134 *CsbHLHs* were identified in the CSS genome^31^. The physical and chemical properties of the 134 CsbHLH proteins were predicted. As shown in Table S1, all of the identified proteins encoded 146–1038 amino acids. Their MWs and theoretical pIs ranged from 16.36 kDa to 114.15 kDa and 4.72 to 9.67, respectively. Four CsbHLHs were likely stable (instability index < 40). The spliced sequences in the CCS genome were not clustered on any chromosomes^31^, and the 134 *CsbHLHs* were renamed *CsbHLH001* to *CsbHLH134* based on the gene ID order (Table S1).

**Figure 1:**
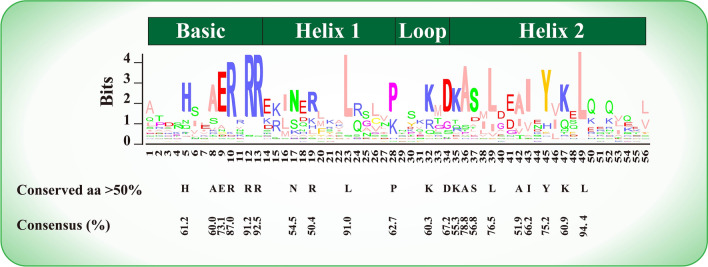
Conserved residue analysis of bHLH domains. The height of each residue indicates the conservation rate. The black letters represent the residues with a consensus ratio greater than 50%.

The conserved domains of the CsbHLH proteins were determined via multiple sequence alignment. As shown in Fig. 1, the bHLH domains possessed four conserved regions: basic, first helix, loop and second helix regions. Twenty-one amino acid residues of the bHLH domain were conserved with a consensus ratio greater than 50%: six residues (His-5, Ala-8, Glu-9, Arg-10, Arg-12 and Arg-13) in the basic region; four residues (Asn-17, Arg-19, Leu-23 and Pro-28) in the helix 1 region; two residues (Lys-32 and Asp-34) in the loop region; and nine residues (Lys-35, Ala-36, Ser-37, Leu-39, Ala-42, Ile-43, Tyr-45, Lys-47 and Leu-49) in the helix 2 region. Arg-12, Arg-13, Leu-23 and Leu-49 were highly conserved (Fig. 1). Additionally, the basic region was absent in CsbHLH067, while the loop and helix 2 regions were not present in CsbHLH056 (Fig. S1).

### Phylogenetic tree analysis of *CsbHLH* proteins

**Figure 2:**
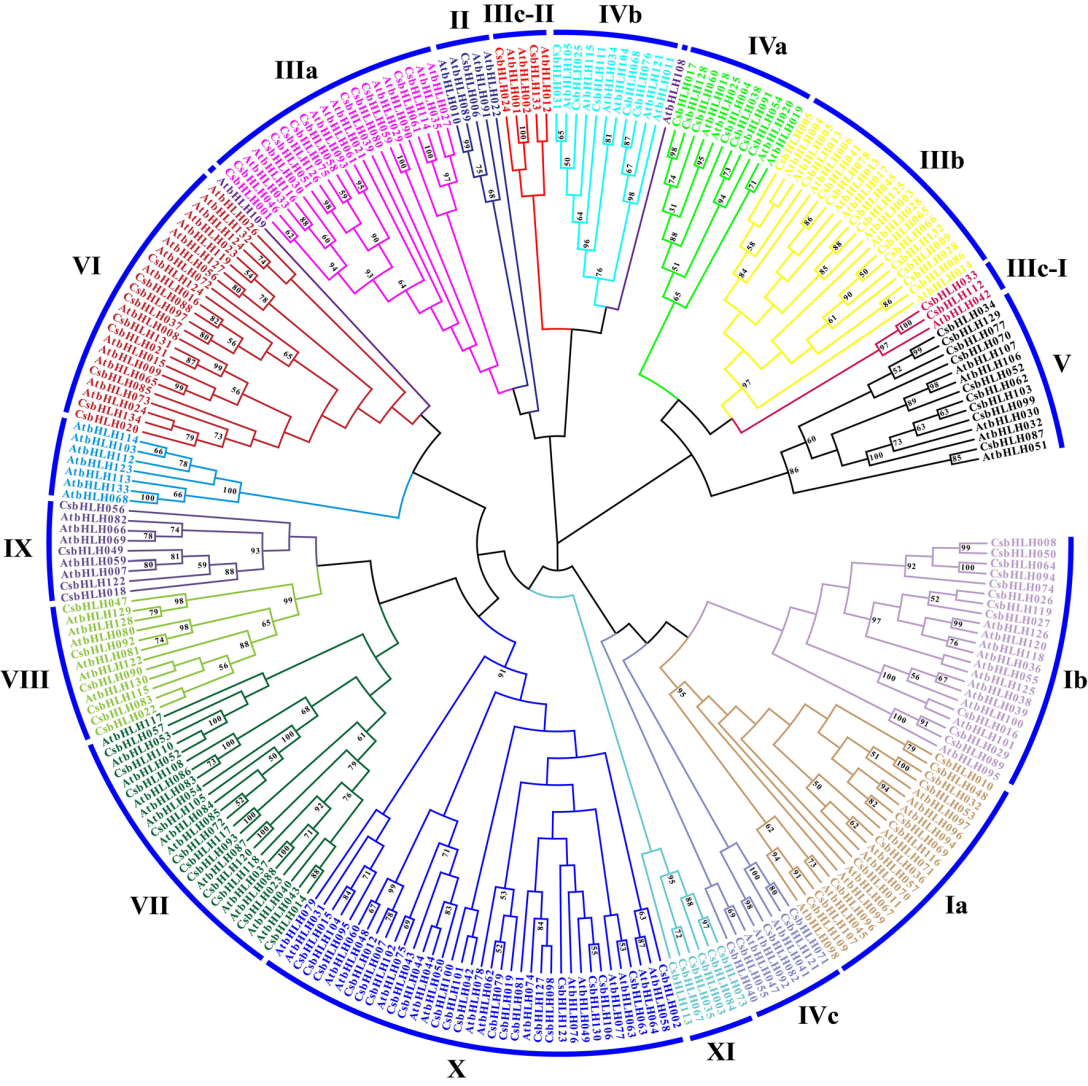
Phylogenetic tree analysis of tea plant and *Arabidopsis thaliana* bHLH proteins. Differently colored branches indicate different subgroups. The black Roman numerals indicate the subgroup name of each branch. Two branches did not include CsbHLH members, and they were not included in the subgroups.

A neighbor-joining phylogenetic tree including all bHLH proteins identified in tea plants and those from *Arabidopsis thaliana* was constructed for the classification of CsbHLH proteins. The CsbHLH proteins were divided into 17 subgroups according to the classification in *Arabidopsis thaliana*^64^ (Fig. 2). Subgroup IIIc was subdivided into subgroups IIIc-I and IIIc-II. The numbers of AtbHLHs and CsbHLHs in each subgroup are listed in Table S2. The members of subgroup II included one CsbHLH and four AtbHLHs. Subgroup X contained the largest numbers of CsbHLHs (21) and AtbHLHs (16). The difference between the members of the CsbHLHs and AtbHLHs within the same group might have resulted from unequal duplication of the bHLH family during species differentiation. To clarify whether the members of 17 subgroups have distinctions in monocots and dicots, all the bHLH proteins of tea plants were used to construct a neighbor-joining phylogenetic tree with those of *Arabidopsis thaliana*, *Oryza sativa* L. and *Actinidia chinensis* (Fig. S2). The results indicated that the members in subgroups IIIc-I and XI were specific to dicots, while the other subgroup included the members of monocots and dicots.

TFs classified in the same group in the phylogenetic tree might possess similar functions. Some *Arabidopsis thaliana* bHLH TFs related to trichome formation were identified, including Transparent Testa8 (TT8)^65^, EGL3^66^, GL3^25^, and Myelocytomatosis1 (MYC1)^67^. They were mapped to AtbHLH042, AtbHLH001, AtbHLH002 and AtbHLH012, respectively. All of these TFs were included in subgroups IIIc-I and IIIc-II (Fig. 2). Therefore, subgroups IIIc-I and IIIc-II were defined as ‘trichome-related groups’, and their members might be involved in tea trichome formation.

In addition, the conserved motifs and gene structures of the *CsbHLHs* were analyzed. Information on 15 identified motifs is listed in Table S3. The results showed that members of the same group might possess similar motifs and gene structures (Fig. S3).

### Transcriptome analysis of *CsbHLHs* in different developmental leaf tissues in tea plants

**Figure 3:**
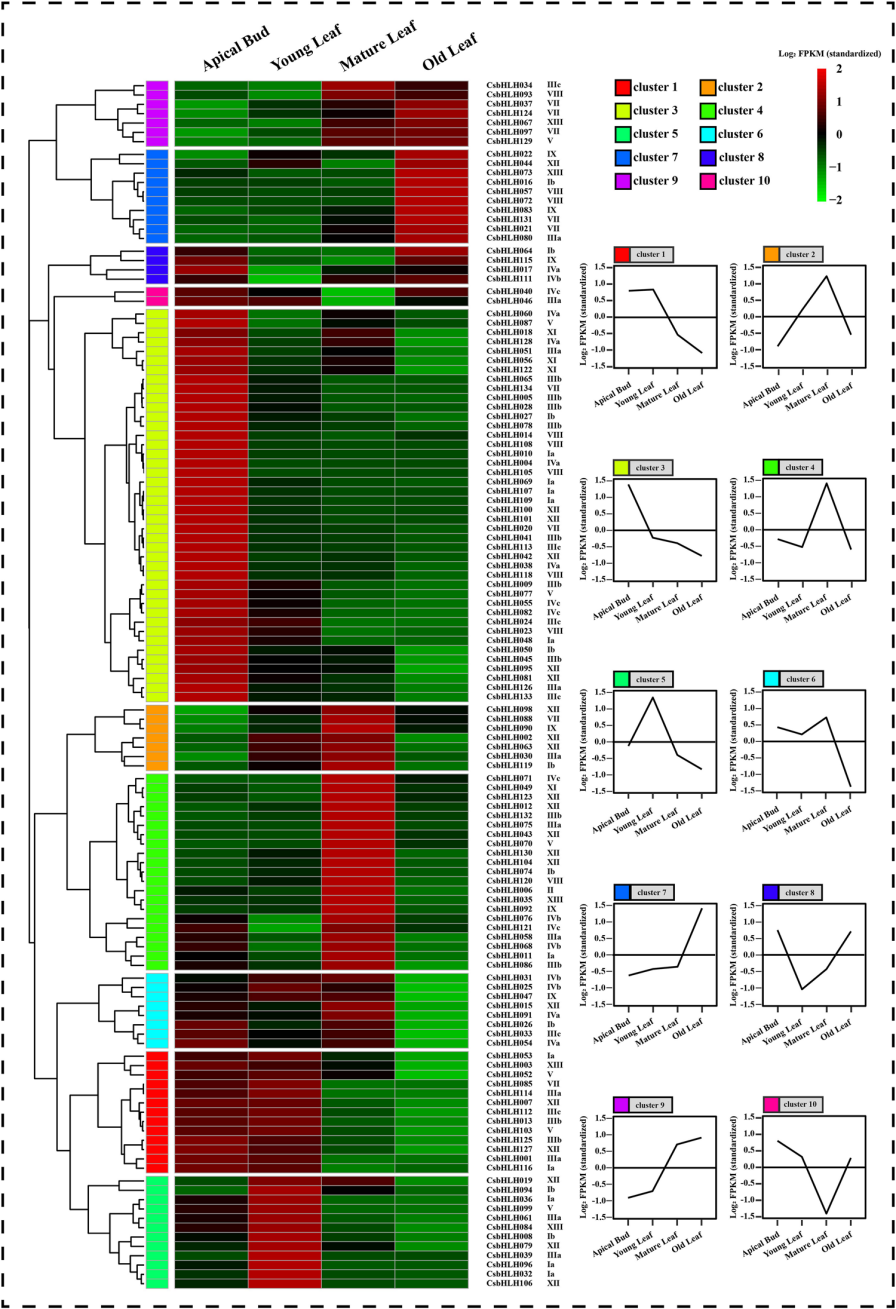
Transcriptome analysis of *CsbHLHs* in different developmental leaf tissues. The name of each gene and the short name of the phylogenetic group are listed on the right of the heatmap. Line charts were generated using the mean value for the whole cluster. Log2 values of fragments per kilobase of exon per million fragments mapped (FPKM) were used to construct the heat map according to the hierarchical clustering analysis.

Leaf trichomes are distributed mainly in the apical buds and young leaves. To further understand the potential function of CsbHLH proteins during leaf trichome formation in tea plants, the expression patterns of *CsbHLHs* in different developmental leaf tissues were determined, including apical buds, young leaves, mature leaves and old leaves. The RNA-seq data of *CsbHLHs* in different developmental leaf tissues were downloaded from TPIA^31^. Eight *CsbHLHs* (*CsbHLH029*, *CsbHLH059*, *CsbHLH062*, *CsbHLH066*, *CsbHLH089*, *CsbHLH102*, *CsbHLH110* and *CsbHLH117*) might be transcribed at low levels in the different developmental leaf tissues, which could not be quantified. According to the similarity of the observed expression patterns, the heatmap was hierarchically clustered into 10 clusters (Fig. 3). The expression patterns of *CsbHLHs* in clusters 1, 3 and 5 were consistent with the distribution of tea leaf trichomes and primarily associated with apical buds and young leaves. *CsbHLHs* in clusters 2, 4, 6, 7 and 9 were highly expressed in the mature and old leaves of tea plants, while the expression of *CsbHLHs* in clusters 8 and 10 was high in the apical buds and old leaves. The members of clusters 1, 3 and 5 might be involved in trichome formation in tea plants.

Additionally, trichome formation is closely related to root hair formation in plants. In *Arabidopsis thaliana*, an MBW transcriptional activator complex can promote trichome formation and inhibit root hair formation by inducing *GL2* expression^47^. Therefore, the expression profiles of *CsbHLHs* of cluster 1, cluster 3 and cluster 5 in eight different tissues, including the apical buds, young leaves, mature leaves, old leaves, root, flower, fruit and stem, were investigated. The RNA-seq data of *CsbHLH*s in eight different tissues were downloaded from TPIA^31^. The results showed that high expression of *CsbHLHs* was observed in the tender tissues (apical buds and young leaves), while low expression was observed in the roots of tea plants (Fig. S4).

### Expression patterns of *CsbHLHs* in different developmental leaf tissues in tea plants

**Figure 4:**
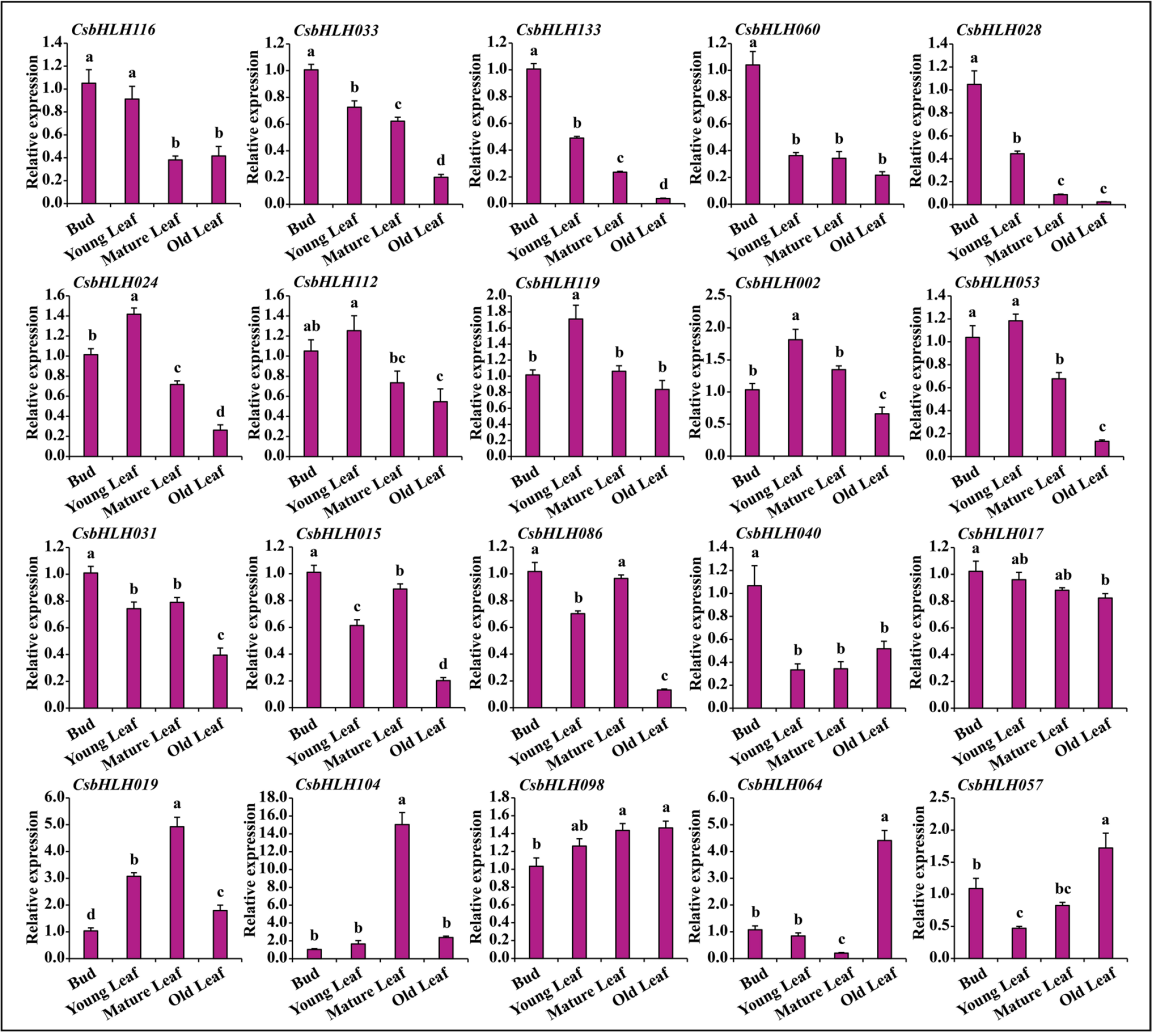
Expression patterns of *CsbHLHs* in different developmental leaf tissues in the cultivar ‘FHDC’. The different letters in the figures indicate significantly different values (P < 0.05, Tukey’s test). For all developmental leaf tissues, three biological replicates and three technical replicates of each biological replicate were performed.

To verify the expression patterns of *CsbHLHs* in different developmental leaf tissues, twenty *CsbHLHs* were analyzed using qRT-PCR. The expression of *CsbHLH116*, *CsbHLH033*, *CsbHLH133*, *CsbHLH060*, *CsbHLH028* and *CsbHLH040* was upregulated in apical bud tissue, while the expression of *CsbHLH024*, *CsbHLH112*, *CsbHLH119*, *CsbHLH002* and *CsbHLH053* exhibited a peak in young leaf tissue (Fig. 4). The expression of all of them decreased with leaf maturation, which was similar to the distribution of tea trichomes. Comprehensive and systematic analysis of the topology of the phylogenetic tree and the expression pattern indicated that *CsHLH024* and *CsbHLH133* were likely candidates for the regulation of trichome formation in tea plants.

### Expression patterns of *CsbHLH024* and *CsbHLH133* in different tea plant cultivars

**Figure 5:**
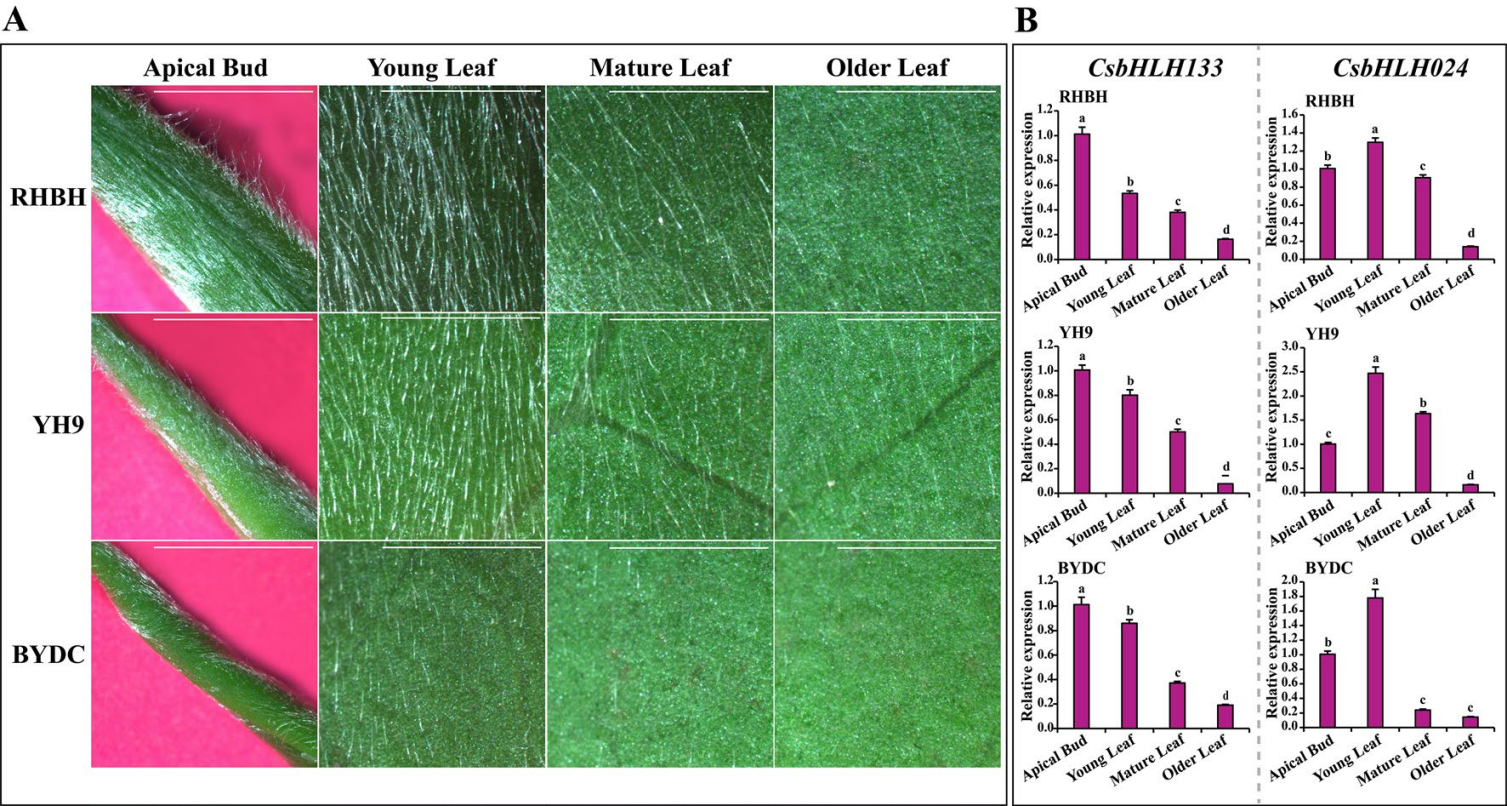
The trichome distribution and expression patterns of *CsbHLH024* and *CsbHLH133* in different tea plant cultivars. A. The trichome distribution in cultivars ‘RHBH’, ‘YH9’ and ‘BYDC’. Scale bars = 5 mm. B. The expression patterns of *CsbHLH024* and *CsbHLH133* in different developmental leaf tissues of different tea plant cultivars. The different letters in the figures indicate significantly different values (P < 0.05, Tukey’s test). For all samples, three biological replicates and three technical replicates of each biological replicate were performed.

To further understand the distribution of leaf trichomes, leaves of different tea plant cultivars were observed using stereoscopy. As shown in Fig. 5A, the tender tissues (apical buds and young leaves) showed more attached trichomes than the mature tissues (mature leaves and old leaves). To understand the expression patterns of *CsbHLH024* and *CsbHLH133* in different developmental leaf tissues of different tea plant cultivars, their transcriptional levels were determined using qRT-PCR. As shown in Fig. 5B, the expression patterns of *CsbHLH133* and *CsbHLH024* maintained good agreement with the trichome distribution in different tea plant cultivars. *CsbHLH133* was highly expressed in apical bud tissue, while *CsbHLH024* expression was upregulated in young leaf tissue. Moreover, the expression of *CsbHLH133* and *CsbHLH024* decreased with the degree of leaf senescence in different tea plant cultivars, which was similar to the trichome distribution in tea plants.

### CsbHLH133 and CsbHLH024 act as transcriptional activators

**Figure 6:**
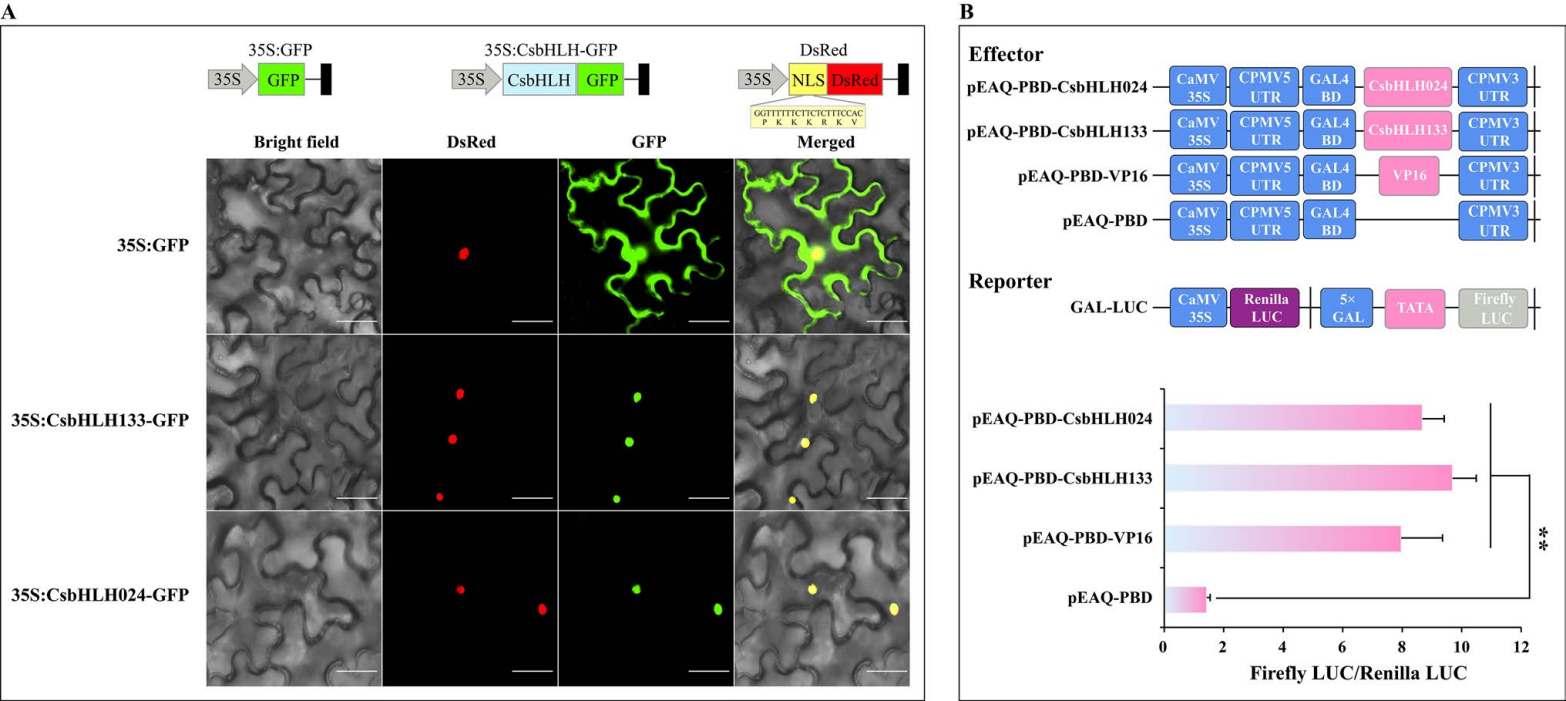
Transcriptional activity of CsbHLH133 and CsbHLH024. (**A**) The subcellular location of *CsbHLH133* and *CsbHLH024* in *Nicotiana benthamiana*. Scale bars = 50 μm. (**B**) The transcriptional activation of CsbHLH133 and CsbHLH024 in *planta*. The data are presented as the means ± SDs (n = 7). Empty vector (PBD) and pBD-VP16 were used as negative and positive controls, respectively. Significant differences were determined using Student’s t-test by comparison to the negative control (**, P < 0.01).

To verify whether CsbHLH133 and CsbHLH024 have transcriptional activation ability, subcellular location and transcriptional activation assays were performed. As shown in Fig. 6A, fluorescence signals from the empty vector were located in the cell nucleus and cytoplasm, while those of the 35S:CsbHLH133-GFP and 35S:CsbHLH024-GFP proteins were found in the cell nucleus. These results indicated that *CsbHLH133* and *CsbHLH024* were localized in the cell nucleus. A dual-luciferase reporter assay showed that CsbHLH024 and CsbHLH133 could strongly enhance the activity of the reporter. The results confirmed that CsbHLH024 and CsbHLH133 served as activators with transcriptional activity in *planta* (Fig. 6B).

### Potential protein regulatory network of trichome formation

**Figure 7:**
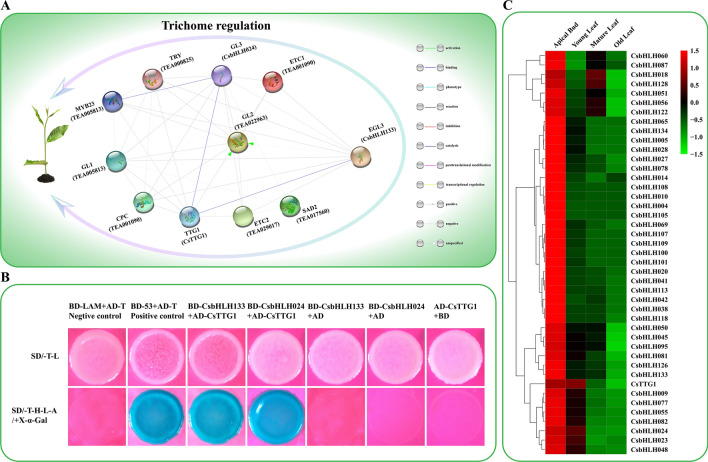
Functional protein association network of trichome formation and a Y2H assay. (**A**) The potential tea trichome regulatory network. A functional protein association network was constructed based on the TFs associated with *Arabidopsis thaliana* trichome formation. Homologous genes were found in the CSS genome, including *CsbHLH024* and *CsbHLH133*. The name of homologous CsbHLH is shown in brackets under *Arabidopsis thaliana*. (**B**) Yeast two-hybrid assay of protein–protein interactions between CsbHLH024 or CsbHLH133 and CsTTG1. BD and AD represent empty pGBKT7 and pGADT7 vectors, respectively. SD/-T-L: synthetic dextrose medium lacking tryptophan and leucine; SD/-T-H-L-A: synthetic dextrose medium lacking tryptophan, histidine, leucine and adenine. Positive bacteria were stained using X-α-Gal. C. The expression patterns of *CsTTG1* and the members of cluster 3 in different developmental leaf tissues. The name of each gene is listed on the right side of the heatmap. Log2 values of FPKM were used to construct the heat map.

bHLH TFs usually interact with other TFs to regulate plant growth and development. A potential functional protein association network in tea plants was constructed based on the regulation of *Arabidopsis thaliana* trichome formation by multiple TFs, including MYB23, TRY, GL3, ETC1, GL1, GL2, EGL3, SAD2, ETC2, TTG1 and CPC^63^ (Fig. 7A). The network showed that CsbHLH024 and CsbHLH133 were likely to bind to multiple TFs. A Y2H assay indicated that CsbHLH024 and CsbHLH133 could interact with CsTTG1, which is a regulator of tea trichome formation (Fig. 7B). In addition, the expression of *CsTTG1* maintained good agreement with that of *CsbHLH024* and *CsbHLH133* (Fig. 7C). These results suggested that CsbHLH024 and CsbHLH133 might regulate trichome formation by interacting with multiple TFs.

## Discussion

The identification of gene family members has been widely performed in many plants, and it has contributed to identifying gene functions^68–70^. Trichomes were conducive not only to plant resistance but also to tea flavor and aroma^32^. Numerous studies have demonstrated that bHLH TFs contribute to trichome formation^45–50^. However, whether bHLH TFs are involved in tea trichome formation is still unknown. In this study, genome-wide identification of the tea plant *bHLH* family was systematically and comprehensively performed. This study provides a further understanding of the relationship between candidate *bHLH* genes and trichome formation.

A total of 134 *CsbHLH* genes were identified in the CSS genome. The different plant species possessed different numbers of bHLH members, which ranged from 85 to 319^71,72^. Members of the bHLH family were identified in *Ginkgo biloba* (85)^71^, *Solpinganum tuberosum* L. (124)^73^, *Solanum lycopersicum* (159)^74^, *Oryza sativa* L. (167)^75^, *Malus pumila* (188)^76^ and *Glycine max* (319)^72^. Twenty-one amino acid residues were conserved in the bHLH domain of tea plants with a consensus rate greater than 50% (Fig. 1), as observed in previous studies^38,77^. Glu-13 and Arg-16 (according to Glu-9 and Arg-12 in our alignment) could bind to the E-box; His-9, Glu-13 and Arg-17 (according to His-5, Glu-9 and Arg-13 in our alignment) could recognize the G-box^39,40,78^; Glu-13 and Arg-17 (according to Glu-9 and Arg-13 in our alignment) were important for DNA binding, and Leu-27 (according to Leu-23 in our alignment) played a vital role in dimerization activity in the bHLH domain^79,80^.

All CsbHLH proteins were divided into 17 subgroups according to the *Arabidopsis thaliana* classification^64^. Members of the same group in the phylogenetic tree might possess similar functions. *INDUCER OF CBF EXPRESSION1 (ICE1)* and *INDUCER OF CBF EXPRESSION2 (ICE2)* were related to the cold acclimation response and freezing tolerance in *Arabidopsis thaliana*^81,82^. They were mapped to *AtbHLH116* and *AtbHLH033* and located in subgroup IIIa. *CsICE1* might be involved in the ICE1-C-repeat binding factor (CBF) cold response pathway in tea plants^83^, which was mapped to *CsbHLH001* and classified into subgroup IIIa. *FER-LIKE IRON DEFICIENCY-INDUCED TRANSCRIPTION FACTOR (FIT)* was mapped to *AtbHLH029* and included in subgroup IIIa. It was responsive to iron deficiency in *Arabidopsis thaliana* roots^84^. The members of subgroup IIIa were likely to be involved in the response to abiotic stress. The *bHLHs* associated with *Arabidopsis thaliana* trichome formation were contained in subgroups IIIc-I and IIIc-II (Fig. 2). These two subgroups were defined as ‘trichome-related groups’ in this study. *CsbHLH024* and *CsbHLH133,* the homologs of *GL3* and *EGL3* in *Arabidopsis thaliana*, belonged to ‘trichome-related groups’ (Fig. 2). In addition, the members of the same group exhibited similar gene structures and motifs (Fig. S4), which also indicated that the genes within the same group might play similar roles.

Expression pattern analysis facilitated the understanding of gene function. The expression patterns of clusters 1, 3 and 5 were in agreement with the tea trichome distribution (Fig. 3) and focused on tender tissues (apical buds and young leaves). *CsbHLH024* and *CsbHLH133* were divided into cluster 3. Their expression peaked in the tender tissues of different tea plant cultivars, including apical buds and young leaves (Fig. 5B). Therefore, *CsHLH024* and *CsbHLH133* might be associated with trichome formation in tea plants. Moreover, CsHLH024 and CsbHLH133 were located in the cell nucleus (Fig. 6A) and possessed transcriptional activity functions (Fig. 6B). The homologs of CsbHLH024 and CsbHLH133 usually regulate trichome formation by interacting with other TFs in *Arabidopsis thaliana*. *CsTTG1* was involved in tea plant trichome formation, and the overexpression of *CsTTG1* could enhance the trichome density of *Arabidopsis thaliana*^85^; the functions of other Clusters of Orthologous Groups (COGs) (MYB23, TRY, ETC1, GL1, GL2, SAD2, ETC2 and CPC) in trichome formation were less known in tea plants. CsbHLH024 and CsbHLH133 could interact with CsTTG1 in the heterologous system (Fig. 7B). CsbHLH024 and CsbHLH133 might be associated with the regulation of tea plant trichome formation by interacting with CsTTG1.

However, the regeneration rate of tea plant explants in vitro is low because the tea plant is a perennial woody species. Tea plant tissues are rich in the polyphenols. Polyphenols can inhibit the activity of *Agrobacterium tumefaciens*, which results in low efficiency tea plant genetic transformation^86^. A stable genetic transformation system for tea plants is still unavailable and needs further exploration. Thus, the functions of candidate CsbHLH TFs in the regulation of trichome formation must be further addressed using multiple methods.

## Conclusions

In total, 134 CsbHLH proteins were identified in the CSS genome. Phylogenetic tree, gene structure and protein motif analyses of these proteins were conducted in this study. All CsbHLH proteins were divided into 17 subgroups. Subgroups IIIc-I and IIIc-II were defined as the ‘trichome-related groups’, and their members were likely to be associated with trichome formation. The members of clusters 1, 3 and 5 were candidates for trichome formation in tea plants. Notably, *CsbHLH024* and *CsbHLH133* classified into the ‘trichome-related group’ and included in cluster 3 were highly expressed in the tender tissues of different tea plant cultivars. The expression of *CsbHLH024* and *CsbHLH133* was similar to the trichome distribution in tea plants. In addition, *CsbHLH024* and *CsbHLH133* were located in the cell nucleus. They possessed transcriptional activation ability and might control trichome formation by interacting with CsTTG1. This study provides useful information for the further study of CsbHLH TF function in the regulation of trichome formation in tea plants.

## Supplementary Information

Supplementary Information 1.

Supplementary Information 2.

## Data Availability

Most data generated or analyzed during this study are included in this article and its supplemental files. The sequencing data used and analyzed during this study are available in the TPIA database (http://tpia.teaplant.org/index.html).

## Abbreviations

bHLH: Basic helix-loop-helix
TFs: Transcription factors
CSS: *Camellia sinensis* Var. *sinensis*
Y2H: Yeast two-hybrid
WDR: WD40-repeat
MBW: MYB-BHLH-WDR
HLH: Helix-loop-helix
GL3: GLABRA3
EGL3: ENHANCER OF GLABRA3
GL2: GLABRA2
FRC4: FURCA4
TPIA: Tea plant information archive
NCBI: National Center for Biotechnology Information
HMM: Hidden Markov model
Mws: Molecular weights
pIs: Isoelectric points
MEME: Multiple EM for Motif Elicitation
CDSs: Coding DNA sequences
TAIR: The *Arabidopsis* Information Resource
‘FHDC’: ‘Fenghuangdancong’
‘RHBH’: ‘Renhuabaihao’
‘YH9’: ‘Yinghongjiuhao’
‘BYDC’: ‘Baiyedancong’
NLS: Nuclear localization signal
TRY: Triptychon
ETC1: ENHANCER of TRY and CPC1
GL1: GLABRA1
CPC: CAPRICE
TTG1: Transparent TESTA GLABRA1
ETC2: ENHANCER of TRY and CPC2
SAD2: Sensitive to ABA and drought2
TT8: Transparent Testa8
MYC1: Myelocytomatosis1
FPKM: Fragments per kilobase of exon per million fragments mapped
ICE1: Inducer OF CBF expression1
ICE2: Inducer OF CBF expression2
CBF: C-repeat binding factor
FIT: FER-like iron deficiency-induced transcription factor
COGs: Clusters of Orthologous Groups

## Materials availability

The tea plant cultivars used in this study were cultivated at South China Agricultural University (Guangzhou, China). The rights of these plant materials were obtained by our lab.
