# Supplementary material for: Genome-wide identification of the tea plant bHLH transcription factor family and discovery of candidate regulators of trichome formation
Source: Sci Rep. 2021 May 24;11:10764. doi: 10.1038/s41598-021-90205-7 (PMC8144589; doi:10.1038/s41598-021-90205-7)
Supplement: Supplementary file 1 — Supplementary Information 1. [file 41598_2021_90205_MOESM1_ESM.pdf]

Genome-wide identification of the tea plant bHLH transcription factor family and discovery of candidate regulator of trichome formation
‘Supplemental Information’

Renjian Liu, Yuyuan Wang, Song Tang, Jiarong Cai, Shaoqun Liu, Peng Zheng, Binmei Sun

March 14th, 2021

Contents

Figure S1. The multiple alignment of CsbHLH conserved domain. **3**

Figure S2. Phylogenetic tree of the bHLH family in *Camellia sinensis* L., *Arabidopsis thaliana*, *Actinidia chinensis* and *Oryza sativa* L.. **4**

Figure S3. Conserved motifs and gene structure distributions of CsbHLH gene family. **5**

Figure S4. The expression pattern of CsbHLHs of cluster 1, cluster 3 and cluster 5 in different tissues. **6**

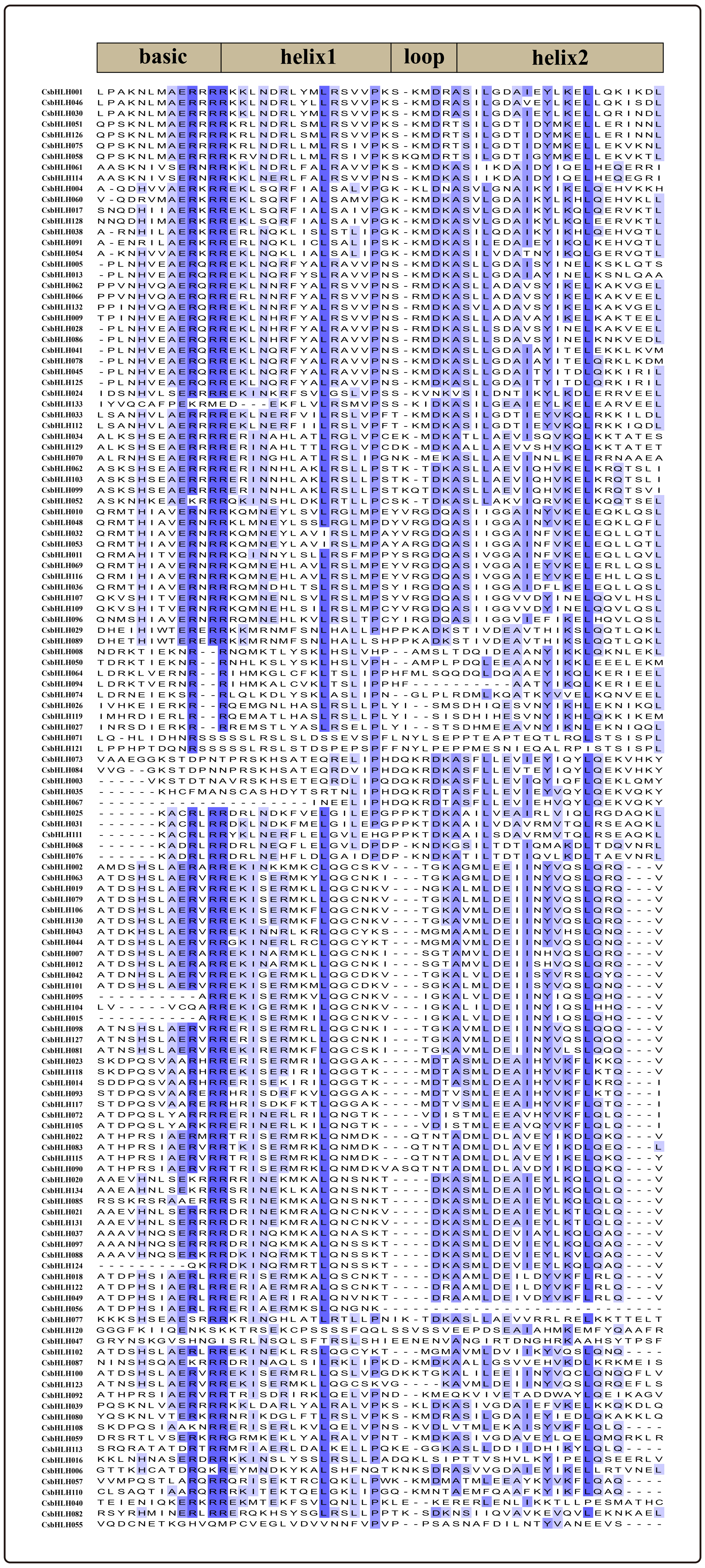

Figure S1. The multiple alignment of CsbHLH conserved domain. The conserved amino acids with more than 33% identify are shown in blue. The conservation ratio of them increased with deepening colour.

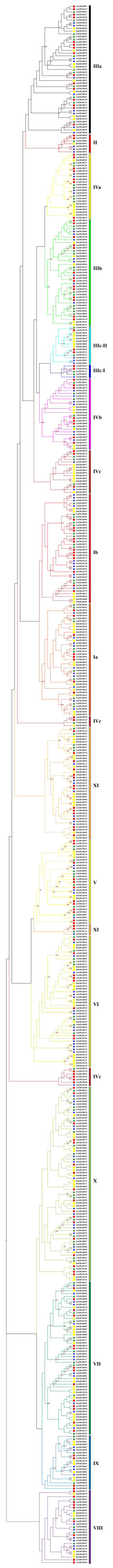

Figure S2. Phylogenetic tree of the bHLH family in *Camellia sinensis* L., *Arabidopsis thaliana*, *Actinidia chinensis* and *Oryza sativa* L.. The different-coloured branches indicated different groups of bHLH proteins.
Red circle, green triangle, yellow rect and blue star represented bHLH proteins in *Actinidia chinensis*, *Camellia sinensis* L. *Oryza sativa* L. and *Arabidopsis thaliana,* respectively.

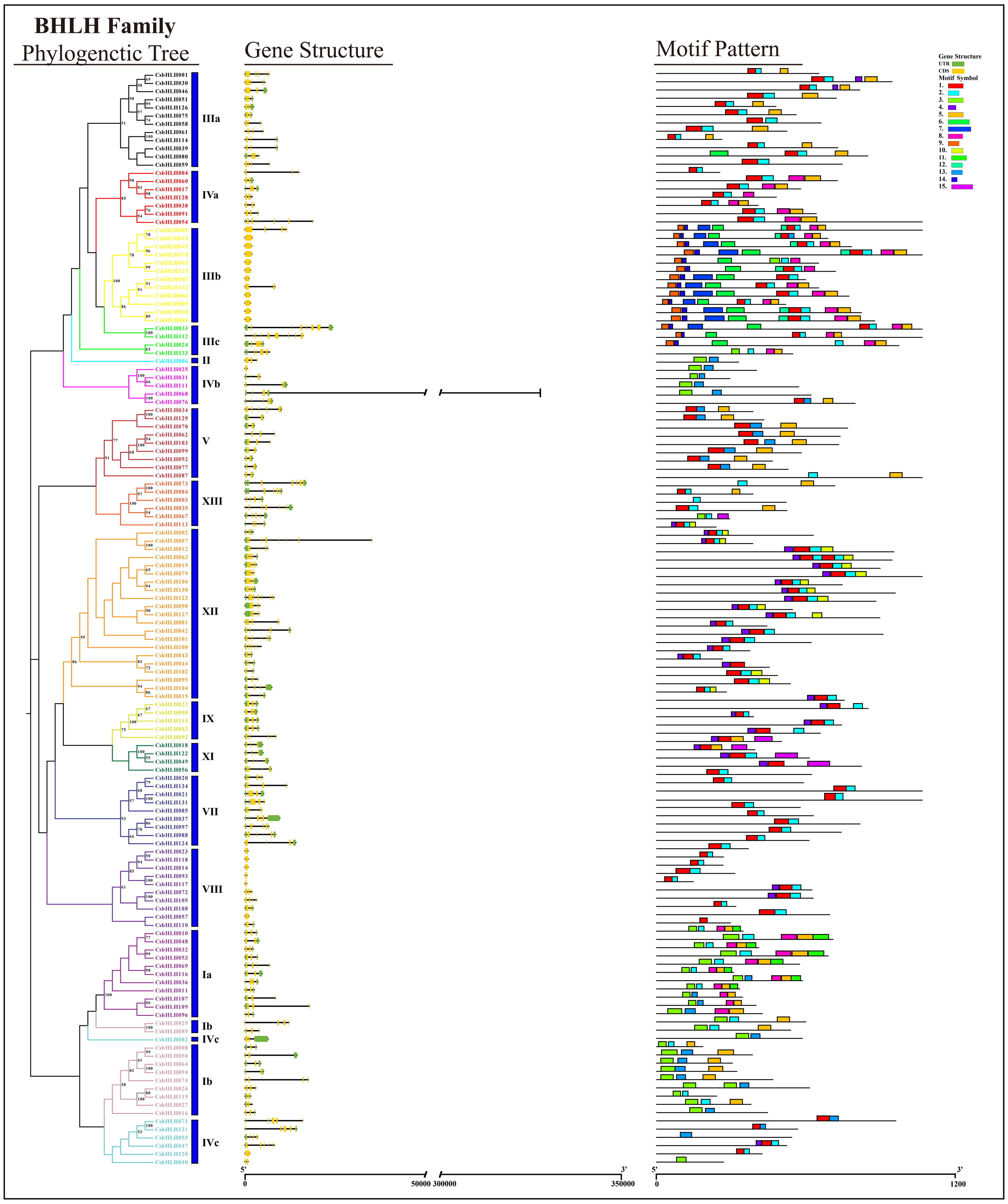

Figure S3. Conserved motifs and gene structure distributions of CsbHLH gene family.
The phylogenetic tree included all identified CsbHLHs was constructed using Neighbor-joining method.
The yellow and green boxes indicated CDS and UTR of *CsbHLHs* (Left).
Different coloured boxes represented different motifs (Right).

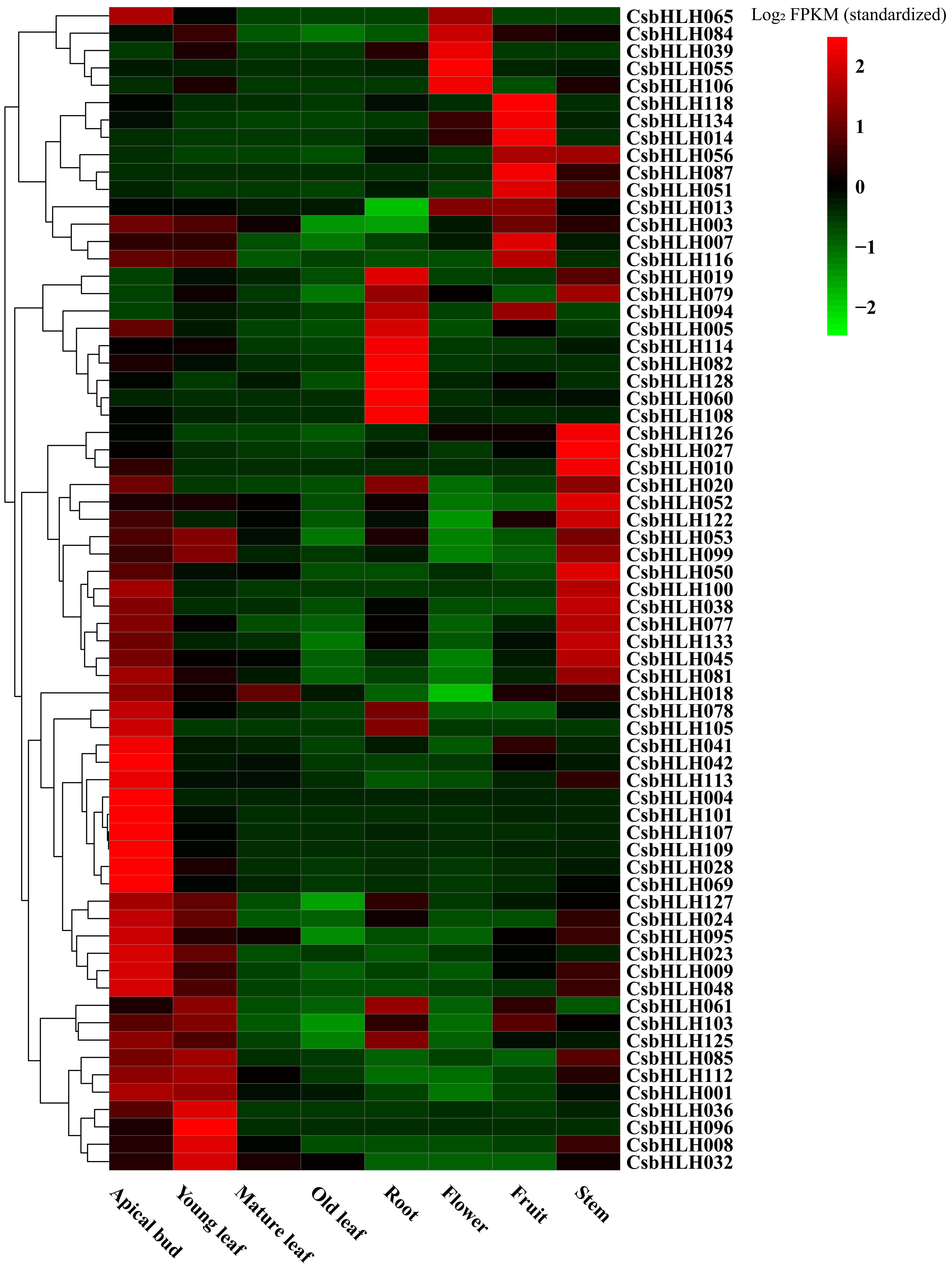

Figure S4. The expression pattern of CsbHLHs of cluster 1, cluster 3 and cluster 5 in different tissues. The RPKM values were downloaded from the Tea Plant Information Archive (TPIA) and used to construct the heat map.
