# Supplementary material for: Genome-wide identification of the tea plant bHLH transcription factor family and discovery of candidate regulators of trichome formation
Source: Sci Rep. 2021 May 24;11:10764. doi: 10.1038/s41598-021-90205-7 (PMC8144589; doi:10.1038/s41598-021-90205-7)
Supplement: Supplementary file 2 — Supplementary Information 2. [file 41598_2021_90205_MOESM2_ESM.docx]

Genome-wide identification of the tea plant bHLH transcription factor family and discovery of candidate regulator of trichome formation

**‘Supplemental Information’**

Renjian Liu, Yuyuan Wang, Song Tang, Jiarong Cai, Shaoqun Liu, Peng Zheng, Binmei Sun

March 14th, 2021

**Contents**

[**Table S1.** The corresponding information of 134 *CsbHLH* genes 3](#_Toc62199583)

[**Table S2.** The number of AtbHLH and CsbHLH in each group 7](#_Toc62199584)

[**Table S3.** The corresponding information of 15 conserved motifs 8](#_Toc62199585)

[**Table S4.** List of bHLH genes in *Arabidopsis thaliana*, *Oryza sativa* L. and *Actinidia chinensis* 9](#_Toc62199586)

[**Table S5.** List of the primers for this study 13](#_Toc62199587)

# **Table S1.** The corresponding information of 134 *CsbHLH* genes

| **Gene name** | **Scaffold** | **Position** | **Length** | **MW** | **pI** | **Instability** | **Corresponding gene ID** |
| --- | --- | --- | --- | --- | --- | --- | --- |
|  |  |  | **(aa)** | **(Da)** |  | **index (II)** | **in CSS** |
| CsbHLH001 | Scaffold1486 | 47414-54088 | 614 | 67133.4 | 6.18 | 48.37 | TEA000036 |
| CsbHLH002 | Scaffold1241 | 663337-665557 | 227 | 25507.98 | 8.31 | 54.70 | TEA000421 |
| CsbHLH003 | Scaffold267 | 2032154-2037094 | 366 | 40679.24 | 5.82 | 63.61 | TEA000472 |
| CsbHLH004 | Scaffold1420 | 779143-794111 | 241 | 26687.34 | 8.88 | 47.64 | TEA000679 |
| CsbHLH005 | Scaffold1796 | 565020-576545 | 1003 | 110270.21 | 5.83 | 49.97 | TEA000833 |
| CsbHLH006 | Scaffold610 | 4087759-4090972 | 516 | 57525.9 | 5.88 | 51.21 | TEA001205 |
| CsbHLH007 | Scaffold610 | 2830560-2865851 | 593 | 67002.81 | 6.28 | 47.15 | TEA001206 |
| CsbHLH008 | Scaffold2325 | 927911-931051 | 177 | 19903.84 | 8.82 | 36.34 | TEA001971 |
| CsbHLH009 | Scaffold2673 | 166688-168157 | 489 | 54437.31 | 6.07 | 50.99 | TEA002159 |
| CsbHLH010 | Scaffold569 | 710799-714056 | 329 | 37023 | 6.41 | 66.20 | TEA002304 |
| CsbHLH011 | Scaffold3382 | 1388654-1391180 | 316 | 35982.9 | 7.17 | 65.36 | TEA002421 |
| CsbHLH012 | Scaffold622 | 1799927-1806240 | 365 | 40487.47 | 5.38 | 53.35 | TEA003290 |
| CsbHLH013 | Scaffold295 | 1573300-1575246 | 648 | 71322.94 | 5.34 | 49.46 | TEA003964 |
| CsbHLH014 | Scaffold3331 | 479823-480584 | 253 | 28873.27 | 5.41 | 75.24 | TEA004704 |
| CsbHLH015 | Scaffold3331 | 207847-213353 | 266 | 29683.08 | 5.83 | 52.55 | TEA004707 |
| CsbHLH016 | Scaffold522 | 120630-123394 | 263 | 29945.09 | 5.86 | 56.88 | TEA004897 |
| CsbHLH017 | Scaffold1077 | 419327-422994 | 341 | 38229.54 | 7.71 | 45.57 | TEA005840 |
| CsbHLH018 | Scaffold1077 | 986330-991168 | 296 | 31695.67 | 5.53 | 57.13 | TEA005852 |
| CsbHLH019 | Scaffold175 | 336831-340003 | 556 | 61436.24 | 8.21 | 47.48 | TEA006010 |
| CsbHLH020 | Scaffold1443 | 1438181-1443017 | 367 | 40650.86 | 5.11 | 61.73 | TEA006216 |
| CsbHLH021 | Scaffold1837 | 611443-616594 | 627 | 68203.1 | 5.93 | 67.13 | TEA007077 |
| CsbHLH022 | Scaffold1579 | 1008242-1011727 | 444 | 49066.1 | 7.21 | 47.34 | TEA007917 |
| CsbHLH023 | Scaffold1444 | 757957-758721 | 218 | 24575.21 | 9.22 | 58.07 | TEA008058 |
| CsbHLH024 | Scaffold3102 | 961833-966938 | 608 | 68593.69 | 5.77 | 57.43 | TEA008168 |
| CsbHLH025 | Scaffold1599 | 565424-566011 | 195 | 21720.83 | 8.24 | 58.19 | TEA008669 |
| CsbHLH026 | Scaffold4000 | 406731-409680 | 362 | 41305.2 | 6.19 | 59.31 | TEA009138 |
| CsbHLH027 | Scaffold4000 | 608339-610303 | 224 | 25562.73 | 5.99 | 53.08 | TEA009147 |
| CsbHLH028 | Scaffold1816 | 3595368-3596822 | 484 | 53276.56 | 5.73 | 48.79 | TEA009193 |
| CsbHLH029 | Scaffold1814 | 893891-906046 | 353 | 38937.14 | 6.87 | 37.22 | TEA009727 |
| CsbHLH030 | Scaffold30 | 3279402-3284931 | 556 | 61241.49 | 5.22 | 57.42 | TEA009785 |
| CsbHLH031 | Scaffold984 | 1176960-1181149 | 237 | 25910.47 | 7.75 | 50.88 | TEA010590 |
| CsbHLH032 | Scaffold946 | 8264-10496 | 313 | 35011.56 | 5.52 | 58.72 | TEA010659 |
| CsbHLH033 | Scaffold3463 | 440717-465099 | 810 | 90450.19 | 5.12 | 66.58 | TEA010741 |
| CsbHLH034 | Scaffold6575 | 714544-724647 | 606 | 68096.92 | 7.88 | 45.04 | TEA011054 |
| CsbHLH035 | Scaffold1185 | 1852229-1865342 | 396 | 43912.06 | 5.82 | 59.71 | TEA011248 |
| CsbHLH036 | Scaffold4138 | 2274408-2277950 | 446 | 49843.22 | 5.66 | 63.97 | TEA011370 |
| CsbHLH037 | Scaffold3537 | 1254907-1264554 | 479 | 53011.4 | 9.26 | 61.00 | TEA011633 |
| CsbHLH038 | Scaffold902 | 403506-406066 | 311 | 35113.75 | 6.33 | 55.37 | TEA011884 |
| CsbHLH039 | Scaffold1277 | 435148-454119 | 553 | 63049.1 | 6.12 | 46.69 | TEA012161 |
| CsbHLH040 | Scaffold338 | 1321514-1322304 | 206 | 23065.49 | 7.74 | 62.17 | TEA012283 |
| CsbHLH041 | Scaffold2527 | 570964-572751 | 595 | 66032.47 | 6 | 42.52 | TEA012449 |
| CsbHLH042 | Scaffold1028 | 389709-402359 | 338 | 38389.4 | 8.53 | 46.10 | TEA012998 |
| CsbHLH043 | Scaffold1028 | 1010135-1012054 | 286 | 32508.71 | 5.76 | 48.03 | TEA013004 |
| CsbHLH044 | Scaffold190 | 327072-329689 | 203 | 22813.58 | 8.59 | 64.32 | TEA013107 |
| CsbHLH045 | Scaffold344 | 2365343-2366830 | 495 | 54855.86 | 6.37 | 45.97 | TEA013248 |
| CsbHLH046 | Scaffold2445 | 1270217-1276186 | 620 | 67984.15 | 6.13 | 42.74 | TEA013512 |
| CsbHLH047 | Scaffold304 | 811235-819349 | 398 | 42958.51 | 8.74 | 54.02 | TEA013610 |
| CsbHLH048 | Scaffold1630 | 42760-46567 | 330 | 37210.88 | 5.15 | 60.48 | TEA013745 |
| CsbHLH049 | Scaffold2753 | 630299-636749 | 286 | 30929.04 | 6.1 | 52.73 | TEA013839 |
| CsbHLH050 | Scaffold5449 | 192965-207432 | 180 | 20241.9 | 6.18 | 47.89 | TEA013874 |
| CsbHLH051 | Scaffold1626 | 64901-67038 | 336 | 37972.97 | 4.85 | 59.82 | TEA013971 |
| CsbHLH052 | Scaffold1562 | 991315-993358 | 271 | 30067.69 | 8.22 | 52.66 | TEA014052 |
| CsbHLH053 | Scaffold1182 | 2049150-2052573 | 321 | 35032.42 | 5.71 | 60.56 | TEA014134 |
| CsbHLH054 | Scaffold2978 | 384549-403375 | 496 | 54242.38 | 6.25 | 52.45 | TEA014288 |
| CsbHLH055 | Scaffold41 | 1183914-1187375 | 253 | 27894.29 | 5.45 | 53.27 | TEA014398 |
| CsbHLH056 | Scaffold4717 | 750739-758002 | 383 | 40107.4 | 5.87 | 40.23 | TEA014689 |
| CsbHLH057 | Scaffold5359 | 477192-478166 | 324 | 36453.55 | 6.71 | 67.79 | TEA014998 |
| CsbHLH058 | Scaffold2611 | 580923-585329 | 308 | 35225.32 | 5.29 | 55.19 | TEA015506 |
| CsbHLH059 | Scaffold740 | 1896571-1903339 | 347 | 39431.14 | 5.91 | 53.64 | TEA015883 |
| CsbHLH060 | Scaffold348 | 1396335-1398507 | 338 | 37317.96 | 5.38 | 56.73 | TEA016075 |
| CsbHLH061 | Scaffold3046 | 523098-528125 | 244 | 27784.48 | 5.36 | 59.69 | TEA016380 |
| CsbHLH062 | Scaffold4289 | 106274-114463 | 357 | 39276.09 | 6.85 | 56.66 | TEA016807 |
| CsbHLH063 | Scaffold1302 | 120378-123778 | 443 | 49334.1 | 6.12 | 60.20 | TEA016938 |
| CsbHLH064 | Scaffold859 | 389726-394023 | 180 | 20577.97 | 9.15 | 31.73 | TEA017105 |
| CsbHLH065 | Scaffold489 | 161302-162360 | 352 | 39603.73 | 5.97 | 54.98 | TEA017239 |
| CsbHLH066 | Scaffold489 | 175595-176959 | 454 | 50793.57 | 5.8 | 52.19 | TEA017243 |
| CsbHLH067 | Scaffold93 | 366901-372913 | 308 | 34179.11 | 5.79 | 56.64 | TEA017390 |
| CsbHLH068 | Scaffold6167 | 800000-1126669 | 336 | 37196.38 | 6.1 | 58.82 | TEA017430 |
| CsbHLH069 | Scaffold1007 | 506154-512919 | 338 | 37462.55 | 7.09 | 61.96 | TEA017853 |
| CsbHLH070 | Scaffold4475 | 185106-187657 | 254 | 27963.76 | 8.18 | 62.29 | TEA017934 |
| CsbHLH071 | Scaffold2493 | 585657-601675 | 564 | 63483.3 | 5.98 | 63.49 | TEA018058 |
| CsbHLH072 | Scaffold1364 | 2060079-2061920 | 367 | 40503.2 | 4.88 | 50.62 | TEA018182 |
| CsbHLH073 | Scaffold943 | 2126217-2143161 | 626 | 68985.98 | 7 | 55.87 | TEA018399 |
| CsbHLH074 | Scaffold2373 | 262610-280206 | 275 | 30000.98 | 5.52 | 41.95 | TEA018698 |
| CsbHLH075 | Scaffold2164 | 708031-709878 | 330 | 37179.41 | 5.46 | 50.12 | TEA018850 |
| CsbHLH076 | Scaffold441 | 141990-149592 | 365 | 40473.34 | 6.64 | 47.36 | TEA019255 |
| CsbHLH077 | Scaffold10260 | 211631-214687 | 274 | 29318.67 | 7.69 | 60.93 | TEA019335 |
| CsbHLH078 | Scaffold3284 | 990002-991852 | 616 | 68150.66 | 6.28 | 43.84 | TEA019380 |
| CsbHLH079 | Scaffold1504 | 351825-354273 | 527 | 57367.03 | 6.34 | 53.49 | TEA021415 |
| CsbHLH080 | Scaffold5970 | 44563-48392 | 490 | 55158.8 | 6.46 | 56.98 | TEA021786 |
| CsbHLH081 | Scaffold1271 | 1618200-1627586 | 518 | 57773.94 | 6.55 | 61.13 | TEA022018 |
| CsbHLH082 | Scaffold6890 | 189579-195970 | 339 | 38617.91 | 6.14 | 64.96 | TEA022030 |
| CsbHLH083 | Scaffold1065 | 1476370-1480171 | 430 | 47454.06 | 6.99 | 52.26 | TEA022347 |
| CsbHLH084 | Scaffold666 | 525626-535853 | 414 | 45950.28 | 8.8 | 52.52 | TEA022528 |
| CsbHLH085 | Scaffold3118 | 481367-485988 | 334 | 36820.06 | 5.91 | 58.00 | TEA022718 |
| CsbHLH086 | Scaffold889 | 530270-531790 | 506 | 56436.96 | 5.75 | 46.24 | TEA022746 |
| CsbHLH087 | Scaffold4521 | 119378-121592 | 306 | 33748.63 | 9.67 | 51.94 | TEA023692 |
| CsbHLH088 | Scaffold953 | 284545-293017 | 429 | 47176.8 | 8.7 | 52.96 | TEA023842 |
| CsbHLH089 | Scaffold9064 | 621395-625297 | 312 | 34626.04 | 8.85 | 46.80 | TEA023960 |
| CsbHLH090 | Scaffold8122 | 209465-212802 | 491 | 53610.18 | 5.79 | 50.12 | TEA023997 |
| CsbHLH091 | Scaffold1984 | 162035-165675 | 371 | 41211.77 | 4.86 | 49.08 | TEA024004 |
| CsbHLH092 | Scaffold1592 | 270548-279129 | 341 | 36582.01 | 5.28 | 56.59 | TEA024017 |
| CsbHLH093 | Scaffold2108 | 82467-82961 | 164 | 18248.66 | 6.96 | 57.48 | TEA024687 |
| CsbHLH094 | Scaffold423 | 3113164-3118264 | 168 | 19012.11 | 9.56 | 30.26 | TEA025381 |
| CsbHLH095 | Scaffold3529 | 449917-453436 | 252 | 27600.83 | 5.3 | 55.28 | TEA025617 |
| CsbHLH096 | Scaffold5270 | 43347-45697 | 221 | 24822.9 | 9.28 | 67.61 | TEA025673 |
| CsbHLH097 | Scaffold460 | 820148-826715 | 423 | 46761.66 | 8.17 | 73.85 | TEA025875 |
| CsbHLH098 | Scaffold3374 | 389879-393983 | 456 | 49137.59 | 5.14 | 49.95 | TEA026346 |
| CsbHLH099 | Scaffold1100 | 526762-529763 | 379 | 42037.1 | 5.97 | 69.01 | TEA026466 |
| CsbHLH100 | Scaffold228 | 4209376-4213826 | 322 | 35878.64 | 6.57 | 47.37 | TEA027045 |
| CsbHLH101 | Scaffold845 | 1635080-1642115 | 471 | 53289.48 | 6.42 | 47.02 | TEA027511 |
| CsbHLH102 | Scaffold171 | 1010242-1012603 | 235 | 26539.63 | 6.45 | 49.45 | TEA027568 |
| CsbHLH103 | Scaffold171 | 623415-630370 | 382 | 42189.21 | 5.97 | 63.18 | TEA027573 |
| CsbHLH104 | Scaffold793 | 546461-553884 | 279 | 30113.66 | 6.53 | 53.63 | TEA028200 |
| CsbHLH105 | Scaffold1047 | 955249-958398 | 326 | 36013.42 | 4.78 | 58.04 | TEA029030 |
| CsbHLH106 | Scaffold1881 | 909732-913166 | 552 | 60173.75 | 5.85 | 54.14 | TEA029062 |
| CsbHLH107 | Scaffold4 | 691332-699752 | 338 | 37375.38 | 5.75 | 64.19 | TEA029105 |
| CsbHLH108 | Scaffold3000 | 507662-509871 | 312 | 34373.13 | 7.13 | 53.01 | TEA029404 |
| CsbHLH109 | Scaffold1523 | 113697-131588 | 391 | 42221.46 | 5.58 | 72.53 | TEA029434 |
| CsbHLH110 | Scaffold3920 | 394224-396647 | 291 | 32845.06 | 4.72 | 59.09 | TEA029491 |
| CsbHLH111 | Scaffold1424 | 349842-361459 | 289 | 31911.1 | 5.32 | 50.27 | TEA029740 |
| CsbHLH112 | Scaffold251 | 4138027-4154130 | 1038 | 114152.6 | 5.06 | 56.95 | TEA029880 |
| CsbHLH113 | Scaffold251 | 3268840-3274354 | 288 | 32500.69 | 6.16 | 50.74 | TEA029953 |
| CsbHLH114 | Scaffold1804 | 429324-438313 | 258 | 29534.11 | 5.16 | 71.31 | TEA030137 |
| CsbHLH115 | Scaffold6238 | 146765-150441 | 381 | 42254.2 | 8.17 | 48.76 | TEA030443 |
| CsbHLH116 | Scaffold1514 | 430597-435336 | 303 | 33567.33 | 8.12 | 59.00 | TEA030725 |
| CsbHLH117 | Scaffold3806 | 99385-99825 | 146 | 16361.6 | 6.5 | 41.49 | TEA030852 |
| CsbHLH118 | Scaffold10805 | 654926-655720 | 264 | 28911.92 | 8.99 | 65.70 | TEA030941 |
| CsbHLH119 | Scaffold2805 | 2083071-2084590 | 237 | 26468.48 | 7.71 | 47.21 | TEA031176 |
| CsbHLH120 | Scaffold3952 | 887080-888324 | 414 | 45602.86 | 6.17 | 54.11 | TEA031802 |
| CsbHLH121 | Scaffold4603 | 593225-607584 | 553 | 62338.59 | 9.39 | 68.44 | TEA031877 |
| CsbHLH122 | Scaffold1316 | 1112582-1117533 | 269 | 29413.25 | 5.92 | 53.30 | TEA032182 |
| CsbHLH123 | Scaffold110 | 102179-110217 | 650 | 70777.41 | 5.93 | 53.70 | TEA032221 |
| CsbHLH124 | Scaffold812 | 224742-238862 | 416 | 44840.29 | 8.3 | 43.83 | TEA032260 |
| CsbHLH125 | Scaffold4224 | 216062-217528 | 488 | 53970.69 | 6.35 | 42.63 | TEA032433 |
| CsbHLH126 | Scaffold3498 | 209084-211393 | 326 | 36873.61 | 4.75 | 51.02 | TEA032613 |
| CsbHLH127 | Scaffold1598 | 1417448-1421340 | 371 | 40529.89 | 5.73 | 51.95 | TEA032897 |
| CsbHLH128 | Scaffold3440 | 346975-348903 | 327 | 36712.35 | 6.23 | 50.50 | TEA033005 |
| CsbHLH129 | Scaffold4449 | 792145-797225 | 264 | 29048.83 | 8.78 | 46.33 | TEA033133 |
| CsbHLH130 | Scaffold2599 | 433302-436135 | 506 | 55195.7 | 6.4 | 49.92 | TEA033198 |
| CsbHLH131 | Scaffold404 | 1159193-1164567 | 723 | 77345.61 | 5.88 | 50.32 | TEA033210 |
| CsbHLH132 | Scaffold4359 | 433068-441351 | 442 | 49393.78 | 6.71 | 46.88 | TEA033639 |
| CsbHLH133 | Scaffold5220 | 1328739-1335553 | 660 | 74288.73 | 5.01 | 44.59 | TEA033721 |
| CsbHLH134 | Scaffold4911 | 113889-125547 | 401 | 43823.94 | 5.18 | 68.36 | TEA033734 |

# **Table S2.** The number of AtbHLH and CsbHLH in each group

| **Group name** | **AtbHLH numbers** | **CsbHLH numbers** | **Total number** |
| --- | --- | --- | --- |
| Ia | 10 | 11 | 21 |
| Ib | 11 | 11 | 22 |
| II | 4 | 1 | 5 |
| IIIa | 9 | 12 | 21 |
| IIIb | 8 | 12 | 20 |
| IIIc-I | 1 | 2 | 3 |
| IIIc-II | 3 | 2 | 5 |
| IVa | 4 | 7 | 11 |
| IVb | 6 | 5 | 11 |
| IVc | 3 | 5 | 8 |
| V | 5 | 9 | 14 |
| VI | 15 | 9 | 24 |
| VII | 13 | 11 | 24 |
| VIII | 6 | 6 | 12 |
| IX | 5 | 4 | 9 |
| X | 16 | 21 | 37 |
| XI | 0 | 6 | 6 |

# **Table S3.** The corresponding information of 15 conserved motifs

|  | **E-value** | **Sites** | **Width** | **Logo** |
| --- | --- | --- | --- | --- |
| 1 | 4.4e-1495 | 98 | 29 | 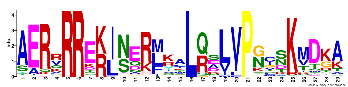 |
| 2 | 8.5e-850 | 100 | 21 | 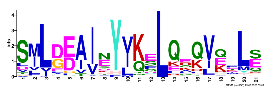 |
| 3 | 2.5e-269 | 33 | 29 | 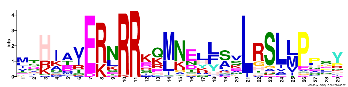 |
| 4 | 1.9e-256 | 32 | 15 | 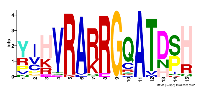 |
| 5 | 8.7e-287 | 63 | 29 | 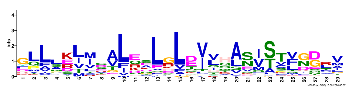 |
| 6 | 1.5e-214 | 17 | 41 | 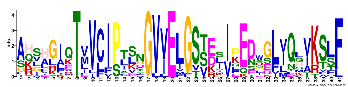 |
| 7 | 1.8e-174 | 11 | 44 | 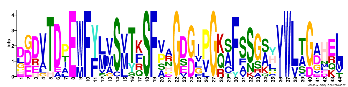 |
| 8 | 1.2e-150 | 33 | 28 | 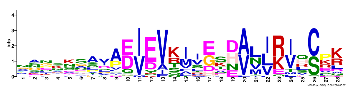 |
| 9 | 1.0e-105 | 16 | 21 | 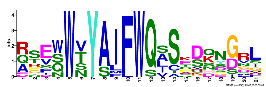 |
| 10 | 2.4e-096 | 15 | 22 | 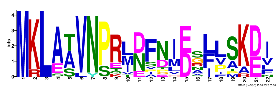 |
| 11 | 1.8e-076 | 8 | 29 | 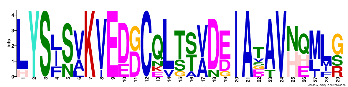 |
| 12 | 5.6e-064 | 7 | 21 | 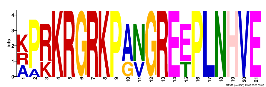 |
| 13 | 3.0e-062 | 29 | 21 | 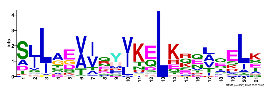 |
| 14 | 1.7e-046 | 16 | 11 | 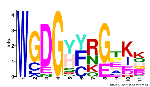 |
| 15 | 1.3e-043 | 5 | 41 | 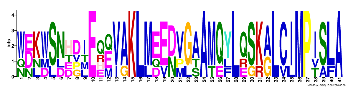 |

# **Table S4.** List of bHLH genes in *Arabidopsis thaliana*, *Oryza sativa* L. and *Actinidia chinensis*

| ***Arabidopsis thaliana*** | | ***Oryza sativa* L.** | | ***Actinidia chinensis*** | |
| --- | --- | --- | --- | --- | --- |
| **ID** | **Gene name** | **ID** | **Gene name** | **ID** | **Gene name** |
| AT3G61950 | AtbHLH067 | BGIOSGA000097-PA | OsbHLH001 | Achn000591 | AcbHLH001 |
| AT3G24140 | AtbHLH097 | BGIOSGA000206-PA | OsbHLH002 | Achn007901 | AcbHLH002 |
| AT2G46810 | AtbHLH070 | BGIOSGA000262-PA | OsbHLH003 | Achn008041 | AcbHLH003 |
| AT5G46690 | AtbHLH071 | BGIOSGA000686-PA | OsbHLH004 | Achn009641 | AcbHLH004 |
| AT4G01460 | AtbHLH057 | BGIOSGA000939-PA | OsbHLH005 | Achn013141 | AcbHLH005 |
| AT5G65320 | AtbHLH099 | BGIOSGA001324-PA | OsbHLH006 | Achn013161 | AcbHLH006 |
| AT1G72210 | AtbHLH096 | BGIOSGA001389-PA | OsbHLH007 | Achn017041 | AcbHLH007 |
| AT1G22490 | AtbHLH094 | BGIOSGA001523-PA | OsbHLH008 | Achn017831 | AcbHLH008 |
| AT5G53210 | AtbHLH098 | BGIOSGA001526-PA | OsbHLH009 | Achn021041 | AcbHLH009 |
| AT3G06120 | AtbHLH045 | BGIOSGA001870-PA | OsbHLH010 | Achn021861 | AcbHLH010 |
| AT1G49770 | AtbHLH095 | BGIOSGA002533-PA | OsbHLH011 | Achn023041 | AcbHLH011 |
| AT1G12540 | AtbHLH055 | BGIOSGA002600-PA | OsbHLH012 | Achn030051 | AcbHLH012 |
| AT1G62975 | AtbHLH125 | BGIOSGA002940-PA | OsbHLH013 | Achn030801 | AcbHLH013 |
| AT4G25410 | AtbHLH126 | BGIOSGA002941-PA | OsbHLH014 | Achn032761 | AcbHLH014 |
| AT4G25400 | AtbHLH118 | BGIOSGA002943-PA | OsbHLH015 | Achn033091 | AcbHLH015 |
| AT5G51790 | AtbHLH120 | BGIOSGA003013-PA | OsbHLH016 | Achn035131 | AcbHLH016 |
| AT5G51780 | AtbHLH036 | BGIOSGA003814-PA | OsbHLH017 | Achn039371 | AcbHLH017 |
| AT2G41240 | AtbHLH100 | BGIOSGA003819-PA | OsbHLH018 | Achn041401 | AcbHLH018 |
| AT5G04150 | AtbHLH101 | BGIOSGA003821-PA | OsbHLH019 | Achn041801 | AcbHLH019 |
| AT3G56970 | AtbHLH038 | BGIOSGA004292-PA | OsbHLH020 | Achn053731 | AcbHLH020 |
| AT3G56980 | AtbHLH039 | BGIOSGA004736-PA | OsbHLH021 | Achn054201 | AcbHLH021 |
| AT2G31210 | AtbHLH091 | BGIOSGA004859-PA | OsbHLH022 | Achn055521 | AcbHLH022 |
| AT2G31220 | AtbHLH010 | BGIOSGA004992-PA | OsbHLH023 | Achn056701 | AcbHLH023 |
| AT1G06170 | AtbHLH089 | BGIOSGA005178-PA | OsbHLH024 | Achn060771 | AcbHLH024 |
| AT4G21330 | AtbHLH022 | BGIOSGA005408-PA | OsbHLH025 | Achn061231 | AcbHLH025 |
| AT2G28160 | AtbHLH029 | BGIOSGA005582-PA | OsbHLH026 | Achn067431 | AcbHLH026 |
| AT2G16910 | AtbHLH021 | BGIOSGA005697-PA | OsbHLH027 | Achn069961 | AcbHLH027 |
| AT5G65640 | AtbHLH093 | BGIOSGA005771-PA | OsbHLH028 | Achn070961 | AcbHLH028 |
| AT5G10570 | AtbHLH061 | BGIOSGA006236-PA | OsbHLH029 | Achn071731 | AcbHLH029 |
| AT1G12860 | AtbHLH033 | BGIOSGA006748-PA | OsbHLH030 | Achn075131 | AcbHLH030 |
| AT3G26744 | AtbHLH116 | BGIOSGA006856-PA | OsbHLH031 | Achn084581 | AcbHLH031 |
| AT1G10610 | AtbHLH090 | BGIOSGA007229-PA | OsbHLH032 | Achn084621 | AcbHLH032 |
| AT5G57150 | AtbHLH035 | BGIOSGA007247-PA | OsbHLH033 | Achn087061 | AcbHLH033 |
| AT4G29930 | AtbHLH027 | BGIOSGA008112-PA | OsbHLH034 | Achn087151 | AcbHLH034 |
| AT4G16430 | AtbHLH003 | BGIOSGA008398-PA | OsbHLH035 | Achn087871 | AcbHLH035 |
| AT1G01260 | AtbHLH013 | BGIOSGA008576-PA | OsbHLH036 | Achn088091 | AcbHLH036 |
| AT2G46510 | AtbHLH017 | BGIOSGA008846-PA | OsbHLH037 | Achn088391 | AcbHLH037 |
| AT4G00870 | AtbHLH014 | BGIOSGA008903-PA | OsbHLH038 | Achn090241 | AcbHLH038 |
| AT1G32640 | AtbHLH006 | BGIOSGA009202-PA | OsbHLH039 | Achn092921 | AcbHLH039 |
| AT4G17880 | AtbHLH004 | BGIOSGA009622-PA | OsbHLH040 | Achn096211 | AcbHLH040 |
| AT5G46760 | AtbHLH005 | BGIOSGA010080-PA | OsbHLH041 | Achn096511 | AcbHLH041 |
| AT5G46830 | AtbHLH028 | BGIOSGA010239-PA | OsbHLH042 | Achn096541 | AcbHLH042 |
| AT4G09820 | AtbHLH042 | BGIOSGA010315-PA | OsbHLH043 | Achn104421 | AcbHLH043 |
| AT4G00480 | AtbHLH012 | BGIOSGA010604-PA | OsbHLH044 | Achn104681 | AcbHLH044 |
| AT5G41315 | AtbHLH001 | BGIOSGA010986-PA | OsbHLH045 | Achn104991 | AcbHLH045 |
| AT1G63650 | AtbHLH002 | BGIOSGA010988-PA | OsbHLH046 | Achn105191 | AcbHLH046 |
| AT2G22770 | AtbHLH020 | BGIOSGA011172-PA | OsbHLH047 | Achn105951 | AcbHLH047 |
| AT2G22760 | AtbHLH019 | BGIOSGA011179-PA | OsbHLH048 | Achn106671 | AcbHLH048 |
| AT2G22750 | AtbHLH018 | BGIOSGA011251-PA | OsbHLH049 | Achn110081 | AcbHLH049 |
| AT4G37850 | AtbHLH025 | BGIOSGA011499-PA | OsbHLH050 | Achn111891 | AcbHLH050 |
| AT3G47640 | AtbHLH047 | BGIOSGA011741-PA | OsbHLH051 | Achn116251 | AcbHLH051 |
| AT4G36060 | AtbHLH011 | BGIOSGA012015-PA | OsbHLH052 | Achn123541 | AcbHLH052 |
| AT3G19860 | AtbHLH121 | BGIOSGA012750-PA | OsbHLH053 | Achn125541 | AcbHLH053 |
| AT5G54680 | AtbHLH105 | BGIOSGA013197-PA | OsbHLH054 | Achn125991 | AcbHLH054 |
| AT1G51070 | AtbHLH115 | BGIOSGA013293-PA | OsbHLH055 | Achn126881 | AcbHLH055 |
| AT3G23210 | AtbHLH034 | BGIOSGA013493-PA | OsbHLH056 | Achn127411 | AcbHLH056 |
| AT4G14410 | AtbHLH104 | BGIOSGA013533-PA | OsbHLH057 | Achn128751 | AcbHLH057 |
| AT5G56960 | AtbHLH041 | BGIOSGA013600-PA | OsbHLH058 | Achn129161 | AcbHLH058 |
| AT5G43650 | AtbHLH092 | BGIOSGA013618-PA | OsbHLH059 | Achn136071 | AcbHLH059 |
| AT1G68810 | AtbHLH030 | BGIOSGA013672-PA | OsbHLH060 | Achn138411 | AcbHLH060 |
| AT3G25710 | AtbHLH032 | BGIOSGA013729-PA | OsbHLH061 | Achn141461 | AcbHLH061 |
| AT3G56770 | AtbHLH107 | BGIOSGA014424-PA | OsbHLH062 | Achn144111 | AcbHLH062 |
| AT2G41130 | AtbHLH106 | BGIOSGA014565-PA | OsbHLH063 | Achn146261 | AcbHLH063 |
| AT2G40200 | AtbHLH051 | BGIOSGA014566-PA | OsbHLH064 | Achn147821 | AcbHLH064 |
| AT1G68240 | AtbHLH109 | BGIOSGA014567-PA | OsbHLH065 | Achn149871 | AcbHLH065 |
| AT1G25310 | AtbHLH108 | BGIOSGA015290-PA | OsbHLH066 | Achn154791 | AcbHLH066 |
| AT3G59060 | AtbHLH065 | BGIOSGA015389-PA | OsbHLH067 | Achn156931 | AcbHLH067 |
| AT2G43010 | AtbHLH009 | BGIOSGA015472-PA | OsbHLH068 | Achn157501 | AcbHLH068 |
| AT1G09530 | AtbHLH008 | BGIOSGA015604-PA | OsbHLH069 | Achn157701 | AcbHLH069 |
| AT4G28800 | AtbHLH056 | BGIOSGA016251-PA | OsbHLH070 | Achn158001 | AcbHLH070 |
| AT4G28815 | AtbHLH127 | BGIOSGA016427-PA | OsbHLH071 | Achn159461 | AcbHLH071 |
| AT4G28811 | AtbHLH119 | BGIOSGA016651-PA | OsbHLH072 | Achn159561 | AcbHLH072 |
| AT4G28790 | AtbHLH023 | BGIOSGA017025-PA | OsbHLH073 | Achn162841 | AcbHLH073 |
| AT2G20180 | AtbHLH015 | BGIOSGA017139-PA | OsbHLH074 | Achn165391 | AcbHLH074 |
| AT2G46970 | AtbHLH124 | BGIOSGA017200-PA | OsbHLH075 | Achn169871 | AcbHLH075 |
| AT3G62090 | AtbHLH132 | BGIOSGA017471-PA | OsbHLH076 | Achn171301 | AcbHLH076 |
| AT1G02340 | AtbHLH026 | BGIOSGA018982-PA | OsbHLH077 | Achn174051 | AcbHLH077 |
| AT5G61270 | AtbHLH072 | BGIOSGA019157-PA | OsbHLH078 | Achn174141 | AcbHLH078 |
| AT4G00050 | AtbHLH016 | BGIOSGA019218-PA | OsbHLH079 | Achn176631 | AcbHLH079 |
| AT5G67110 | AtbHLH073 | BGIOSGA020126-PA | OsbHLH080 | Achn177021 | AcbHLH080 |
| AT4G36930 | AtbHLH024 | BGIOSGA020128-PA | OsbHLH081 | Achn177031 | AcbHLH081 |
| AT3G22100 | AtbHLH117 | BGIOSGA020285-PA | OsbHLH082 | Achn177941 | AcbHLH082 |
| AT1G30670 | AtbHLH052 | BGIOSGA020491-PA | OsbHLH083 | Achn178981 | AcbHLH083 |
| AT2G34820 | AtbHLH053 | BGIOSGA020891-PA | OsbHLH084 | Achn181261 | AcbHLH084 |
| AT3G21330 | AtbHLH087 | BGIOSGA021743-PA | OsbHLH085 | Achn192881 | AcbHLH085 |
| AT5G67060 | AtbHLH088 | BGIOSGA021777-PA | OsbHLH086 | Achn204621 | AcbHLH086 |
| AT3G50330 | AtbHLH037 | BGIOSGA022375-PA | OsbHLH087 | Achn206741 | AcbHLH087 |
| AT5G09750 | AtbHLH043 | BGIOSGA022533-PA | OsbHLH088 | Achn207091 | AcbHLH088 |
| AT4G00120 | AtbHLH040 | BGIOSGA022687-PA | OsbHLH089 | Achn208591 | AcbHLH089 |
| AT1G27740 | AtbHLH054 | BGIOSGA022967-PA | OsbHLH090 | Achn211521 | AcbHLH090 |
| AT2G14760 | AtbHLH084 | BGIOSGA023024-PA | OsbHLH091 | Achn212861 | AcbHLH091 |
| AT4G33880 | AtbHLH085 | BGIOSGA023114-PA | OsbHLH092 | Achn215981 | AcbHLH092 |
| AT1G66470 | AtbHLH083 | BGIOSGA023624-PA | OsbHLH093 | Achn217751 | AcbHLH093 |
| AT5G37800 | AtbHLH086 | BGIOSGA023869-PA | OsbHLH094 | Achn217771 | AcbHLH094 |
| AT2G42280 | AtbHLH130 | BGIOSGA023988-PA | OsbHLH095 | Achn217961 | AcbHLH095 |
| AT1G05805 | AtbHLH128 | BGIOSGA024146-PA | OsbHLH096 | Achn221861 | AcbHLH096 |
| AT2G43140 | AtbHLH129 | BGIOSGA024674-PA | OsbHLH097 | Achn222131 | AcbHLH097 |
| AT1G51140 | AtbHLH122 | BGIOSGA025137-PA | OsbHLH098 | Achn222381 | AcbHLH098 |
| AT4G09180 | AtbHLH081 | BGIOSGA025266-PA | OsbHLH099 | Achn227371 | AcbHLH099 |
| AT1G35460 | AtbHLH080 | BGIOSGA025912-PA | OsbHLH100 | Achn232701 | AcbHLH100 |
| AT3G19500 | AtbHLH113 | BGIOSGA026551-PA | OsbHLH101 | Achn233831 | AcbHLH101 |
| AT3G20640 | AtbHLH123 | BGIOSGA026577-PA | OsbHLH102 | Achn233841 | AcbHLH102 |
| AT4G29100 | AtbHLH068 | BGIOSGA026775-PA | OsbHLH103 | Achn235091 | AcbHLH103 |
| AT2G20095 | AtbHLH133 | BGIOSGA026779-PA | OsbHLH104 | Achn238701 | AcbHLH104 |
| AT4G21340 | AtbHLH103 | BGIOSGA026828-PA | OsbHLH105 | Achn239761 | AcbHLH105 |
| AT4G05170 | AtbHLH114 | BGIOSGA026937-PA | OsbHLH106 | Achn239971 | AcbHLH106 |
| AT1G61660 | AtbHLH112 | BGIOSGA027551-PA | OsbHLH107 | Achn241401 | AcbHLH107 |
| AT4G30980 | AtbHLH069 | BGIOSGA027691-PA | OsbHLH108 | Achn243731 | AcbHLH108 |
| AT2G24260 | AtbHLH066 | BGIOSGA027780-PA | OsbHLH109 | Achn244201 | AcbHLH109 |
| AT5G58010 | AtbHLH082 | BGIOSGA028855-PA | OsbHLH110 | Achn244921 | AcbHLH110 |
| AT1G03040 | AtbHLH007 | BGIOSGA028884-PA | OsbHLH111 | Achn253521 | AcbHLH111 |
| AT4G02590 | AtbHLH059 | BGIOSGA028888-PA | OsbHLH112 | Achn253811 | AcbHLH112 |
| AT1G25330 | AtbHLH075 | BGIOSGA028963-PA | OsbHLH113 | Achn267831 | AcbHLH113 |
| AT1G73830 | AtbHLH050 | BGIOSGA029027-PA | OsbHLH114 | Achn270131 | AcbHLH114 |
| AT1G18400 | AtbHLH044 | BGIOSGA029426-PA | OsbHLH115 | Achn271221 | AcbHLH115 |
| AT4G34530 | AtbHLH063 | BGIOSGA029460-PA | OsbHLH116 | Achn271541 | AcbHLH116 |
| AT1G68920 | AtbHLH049 | BGIOSGA029497-PA | OsbHLH117 | Achn273391 | AcbHLH117 |
| AT1G26260 | AtbHLH076 | BGIOSGA029759-PA | OsbHLH118 | Achn281761 | AcbHLH118 |
| AT5G48560 | AtbHLH078 | BGIOSGA029777-PA | OsbHLH119 | Achn283551 | AcbHLH119 |
| AT3G07340 | AtbHLH062 | BGIOSGA030896-PA | OsbHLH120 | Achn292261 | AcbHLH120 |
| AT1G10120 | AtbHLH074 | BGIOSGA030928-PA | OsbHLH121 | Achn293201 | AcbHLH121 |
| AT3G23690 | AtbHLH077 | BGIOSGA030936-PA | OsbHLH122 | Achn299091 | AcbHLH122 |
| AT2G42300 | AtbHLH048 | BGIOSGA030954-PA | OsbHLH123 | Achn302211 | AcbHLH123 |
| AT3G57800 | AtbHLH060 | BGIOSGA030958-PA | OsbHLH124 | Achn309021 | AcbHLH124 |
| AT1G59640 | AtbHLH031 | BGIOSGA031008-PA | OsbHLH125 | Achn309341 | AcbHLH125 |
| AT5G62610 | AtbHLH079 | BGIOSGA031426-PA | OsbHLH126 | Achn310341 | AcbHLH126 |
| AT4G36540 | AtbHLH058 | BGIOSGA031474-PA | OsbHLH127 | Achn313111 | AcbHLH127 |
| AT2G18300 | AtbHLH064 | BGIOSGA032415-PA | OsbHLH128 | Achn313181 | AcbHLH128 |
|  |  | BGIOSGA032814-PA | OsbHLH129 | Achn313661 | AcbHLH129 |
|  |  | BGIOSGA033493-PA | OsbHLH130 | Achn317751 | AcbHLH130 |
|  |  | BGIOSGA033976-PA | OsbHLH131 | Achn318391 | AcbHLH131 |
|  |  | BGIOSGA035358-PA | OsbHLH132 | Achn321841 | AcbHLH132 |
|  |  | BGIOSGA035596-PA | OsbHLH133 | Achn322811 | AcbHLH133 |
|  |  | BGIOSGA035894-PA | OsbHLH134 | Achn326561 | AcbHLH134 |
|  |  | BGIOSGA035917-PA | OsbHLH135 | Achn329601 | AcbHLH135 |
|  |  | BGIOSGA037460-PA | OsbHLH136 | Achn329831 | AcbHLH136 |
|  |  | BGIOSGA037731-PA | OsbHLH137 | Achn332351 | AcbHLH137 |
|  |  | BGIOSGA037732-PA | OsbHLH138 | Achn333271 | AcbHLH138 |
|  |  | BGIOSGA037733-PA | OsbHLH139 | Achn335591 | AcbHLH139 |
|  |  | BGIOSGA037734-PA | OsbHLH140 | Achn336631 | AcbHLH140 |
|  |  | BGIOSGA037772-PA | OsbHLH141 | Achn338981 | AcbHLH141 |
|  |  | BGIOSGA037775-PA | OsbHLH142 | Achn339261 | AcbHLH142 |
|  |  | BGIOSGA037855-PA | OsbHLH143 | Achn344611 | AcbHLH143 |
|  |  | BGIOSGA040642-PA | OsbHLH144 | Achn346301 | AcbHLH144 |
|  |  |  |  | Achn346471 | AcbHLH145 |
|  |  |  |  | Achn348381 | AcbHLH146 |
|  |  |  |  | Achn348451 | AcbHLH147 |
|  |  |  |  | Achn348741 | AcbHLH148 |
|  |  |  |  | Achn348771 | AcbHLH149 |
|  |  |  |  | Achn353411 | AcbHLH150 |
|  |  |  |  | Achn354401 | AcbHLH151 |
|  |  |  |  | Achn354411 | AcbHLH152 |
|  |  |  |  | Achn354801 | AcbHLH153 |
|  |  |  |  | Achn355681 | AcbHLH154 |
|  |  |  |  | Achn357141 | AcbHLH155 |
|  |  |  |  | Achn359571 | AcbHLH156 |
|  |  |  |  | Achn361761 | AcbHLH157 |
|  |  |  |  | Achn361801 | AcbHLH158 |
|  |  |  |  | Achn367441 | AcbHLH159 |
|  |  |  |  | Achn368981 | AcbHLH160 |
|  |  |  |  | Achn378861 | AcbHLH161 |
|  |  |  |  | Achn379311 | AcbHLH162 |
|  |  |  |  | Achn383051 | AcbHLH163 |
|  |  |  |  | Achn389001 | AcbHLH164 |

# **Table S5.** List of the primers for this study

| **Primer name** | **Sequence** |
| --- | --- |
| qCsbHLH112-F | CTGGATCCAAACCTCGTGCT |
| qCsbHLH112-R | CTGAATGCCCCTCCCATTGT |
| qCsbHLH116-F | AGAACTCGGTGGTGCTTCTG |
| qCsbHLH116-R | CTCCACCGCAATGTGGATCA |
| qCsbHLH053-F | AATACCTCGCCGTCATTCGG |
| qCsbHLH053-R | AGAAGTCGGCGAAAAGCTGA |
| qCsbHLH098-F | ACCTGAGTGTGCCCAGATTG |
| qCsbHLH098-R | CTCCCTGACAAAGTGCACCA |
| qCsbHLH002-F | GCAACAAGTACCACAAGCGG |
| qCsbHLH002-R | AACTCTGCATTGCGAAGGGT |
| qCsbHLH119-F | AGATCCAATCGCGGGTTTCA |
| qCsbHLH119-R | TCTTGCCTTCTTAGGCGCTC |
| qCsbHLH133-F | TCAGCCCATCTGGTTATGCG |
| qCsbHLH133-R | ATCAGTCACGCCAAGCTCAA |
| qCsbHLH024-F | GCCAACCCATCTGGCTATGT |
| qCsbHLH024-R | TACACCCAGCTCAACGACAC |
| qCsbHLH060-F | TTCCGGCGACGATGATTCTT |
| qCsbHLH060-R | GCAACGCCTTTCTGCTTCTC |
| qCsbHLH028-F | TGTTGGGTCCGATGCAATGA |
| qCsbHLH028-R | CATGCTGGCATGGTAAACCG |
| qCsbHLH071-F | TCCTTAGACTTGACGCGAGC |
| qCsbHLH071-R | GTCAACAACGCAGTTGTGCT |
| qCsbHLH104-F | TCTGCCGCTAATCCTTCGTC |
| qCsbHLH104-R | TGCTCCGTAACCGTGGATTC |
| qCsbHLH086-F | TAGTGGCCCTAGGGGTTGAA |
| qCsbHLH086-R | CGCGTCCATTAATCTTGCCG |
| qCsbHLH019-F | GGAACTGAATTGCTTGCCCG |
| qCsbHLH019-R | TTGCGAAAACGTCGTTGGAC |
| qCsbHLH084-F | CAGTGGATTGCGCTGTTACG |
| qCsbHLH084-R | GTGGTGGCTTCTCGAGATCA |
| qCsbHLH106-F | CAGCTCAGAGCAACTTCCCA |
| qCsbHLH106-R | TCTCTGAGCACAACACCGTC |
| qCsbHLH033-F | GGTGTGGTTGAACTTGGCAC |
| qCsbHLH033-R | TTAGAGGTGGACTGCTCGGA |
| qCsbHLH015-F | AGCTTGTCTCGACGAGCATT |
| qCsbHLH015-R | GGTGGCTCATGCGTTTTTGT |
| qCsbHLH031-F | CAACGTCGGTGTGGAACTTG |
| qCsbHLH031-R | GGAACTTGATGCACCACACG |
| qCsbHLH022-F | CCGTCTTCGTGCTCGAGATT |
| qCsbHLH022-R | GGCCAGTGAAACCAGACTCA |
| qCsbHLH057-F | TTGCAGAGTCTTCGTCTCGG |
| qCsbHLH057-R | CCGAGTCTTCTCGCTGATCC |
| qCsbHLH080-F | CCCATGATCCACTGGTTGGT |
| qCsbHLH080-R | CCGGAACCAGAACAGAACGA |
| qCsbHLH064-F | AACTCGATCAAGCTGCCGAA |
| qCsbHLH064-R | ACCACAGGTAGTCTGAGTCCA |
| qCsbHLH017-F | TTGTCGAAAGGCCCTCCAAA |
| qCsbHLH017-R | AGAGGCCGAAACATCAGAGG |
| qCsbHLH111-F | TCTGAGTGATGCTGTTCGCA |
| qCsbHLH111-R | TAGCCTCTGCTTCTCATCGC |
| qCsbHLH034-F | TATTCCGATGGACGCTGACG |
| qCsbHLH034-R | CGTTTTTCACTCGGCTTCCC |
| qCsbHLH124-F | GCCGGTGGAACCTTAGAGTC |
| qCsbHLH124-R | TGGTCAAACCATGGGACGAG |
| qCsbHLH129-F | TGTTGCGAATACACACCCGA |
| qCsbHLH129-R | TCGGGATTGTCGGCATTCAT |
| qCsbHLH040-F | GTGTTTGAGAAGCACGAGGC |
| qCsbHLH040-R | TTCCCAACGTCGTCTCCATC |
| qCsbHLH046-F | CTTTGCCCAGGTTCATTGCC |
| qCsbHLH046-R | TGCAAACCCATTGAAGCAGC |
| SAND1-F | TCCAATTGCCCCCTTAATGACTCA |
| SAND1-R | GTAAGGGCAGGCAAACACCAGGTA |
| CsbHLH133-PEAQ-EGFP-F | ctgcccaaattcgcgaccggtATGTGTTGGGCAATGGCTAA |
| CsbHLH133-PEAQ-EGFP-R | gcccttgctcaccataccggtACACTTGCCAGCAATAGTTG |
| CsbHLH024-PEAQ-EGFP-F | ctgcccaaattcgcgaccggtATGGCTTCTGGGGGGCACCATAA |
| CsbHLH024-PEAQ-EGFP-R | gcccttgctcaccataccggtCTTTCTAACCACTCTCTGAAGG |
| CsbHLH133-PEAQ-PBD-F | gtatcgccgaccggtaggcctATGTGTTGGGCAATGGCTAA |
| CsbHLH133-PEAQ-PBD-R | aaccagagttaaaggcctCTAACACTTGCCAGCAATAG |
| CsbHLH024-PEAQ-PBD-F | gtatcgccgaccggtaggcctATGGCTTCTGGGGGGCACCA |
| CsbHLH024-PEAQ-PBD-R | aaccagagttaaaggcctTCACTTTCTAACCACTCTCT |
| BD-CsbHLH024-F | atggccatggaggccgaattcATGGCTTCTGGGGGGCAC |
| BD-CsbHLH024-R | ccgctgcaggtcgacggatccTCACTTTCTAACCACTCTCTGAAGGG |
| BD-CsbHLH133-F | atggccatggaggccgaattcATGTGTTGGGCAATGGCTAATG |
| BD-CsbHLH133-R | ccgctgcaggtcgacggatccCTAACACTTGCCAGCAATAGTTGAA |
| AD-CsTTG1-F | gccatggaggccagtgaattcATGGAGAATTCGAGCCAAGATTC |
| AD-CsTTG1-R | cagctcgagctcgatggatccTCAAACTTTCAGAAGCTGCATTTT |
